# Spatio-Temporal Simulation of First Pass Drug Perfusion in the Liver

**DOI:** 10.1371/journal.pcbi.1003499

**Published:** 2014-03-13

**Authors:** Lars Ole Schwen, Markus Krauss, Christoph Niederalt, Felix Gremse, Fabian Kiessling, Andrea Schenk, Tobias Preusser, Lars Kuepfer

**Affiliations:** 1 Fraunhofer MEVIS, Bremen, Germany; 2 Computational Systems Biology, Bayer Technology Services, Leverkusen, Germany; 3 Aachen Institute for Advanced Study in Computational Engineering Sciences, RWTH Aachen University, Aachen, Germany; 4 Experimental Molecular Imaging, RWTH Aachen University, Aachen, Germany; 5 School of Engineering and Science, Jacobs University, Bremen, Germany; 6 Institute of Applied Microbiology, RWTH Aachen University, Aachen, Germany; Icahn School of Medicine at Mount Sinai, United States of America

## Abstract

The liver is the central organ for detoxification of xenobiotics in the body. In pharmacokinetic modeling, hepatic metabolization capacity is typically quantified as hepatic clearance computed as degradation in well-stirred compartments. This is an accurate mechanistic description once a quasi-equilibrium between blood and surrounding tissue is established. However, this model structure cannot be used to simulate spatio-temporal distribution during the first instants after drug injection. In this paper, we introduce a new spatially resolved model to simulate first pass perfusion of compounds within the naive liver. The model is based on vascular structures obtained from computed tomography as well as physiologically based mass transfer descriptions obtained from pharmacokinetic modeling. The physiological architecture of hepatic tissue in our model is governed by both vascular geometry and the composition of the connecting hepatic tissue. In particular, we here consider locally distributed mass flow in liver tissue instead of considering well-stirred compartments. Experimentally, the model structure corresponds to an isolated perfused liver and provides an ideal platform to address first pass effects and questions of hepatic heterogeneity. The model was evaluated for three exemplary compounds covering key aspects of perfusion, distribution and metabolization within the liver. As pathophysiological states we considered the influence of steatosis and carbon tetrachloride-induced liver necrosis on total hepatic distribution and metabolic capacity. Notably, we found that our computational predictions are in qualitative agreement with previously published experimental data. The simulation results provide an unprecedented level of detail in compound concentration profiles during first pass perfusion, both spatio-temporally in liver tissue itself and temporally in the outflowing blood. We expect our model to be the foundation of further spatially resolved models of the liver in the future.

## Author Summary

The liver continuously removes xenobiotic compounds from the blood in the mammalian body. Most computational models represent the liver as composed of few well-stirred subcompartments so that a spatially resolved simulation of hepatic perfusion and compound distribution right after drug administration is currently not available. To mechanistically describe the local distribution of compounds in liver tissue during first pass perfusion, we here present a computational model which combines micro-CT based vascular structures with mass transfer descriptions used in physiologically based pharmacokinetic modeling. In the resulting spatio-temporal model, hepatic mass transfer is governed by the physiological architecture and the composition of the connecting hepatic tissue, such that hepatic heterogeneity and spatial distribution can be described mechanistically. The performance of our model is shown for exemplary compounds addressing key aspects of distribution and metabolization of drugs within a mouse liver. We furthermore investigate the impact of steatosis and carbon tetrachloride-induced liver necrosis. Notably, we find that our computational predictions are in qualitative agreement with previous experimental results in animal models. In the future, our spatially resolved model will be extended by including additional physiological information and by taking into account recirculation through the body.

## Introduction

The liver is the main site of metabolization and detoxification of xenobiotics in the body of mammals. Compounds delivered by blood flow through the portal vein and the hepatic artery are continuously processed within hepatic cells, such that foreign and potentially harmful compounds can be cleared from the blood. Metabolization by enzyme-catalyzed biotransformation produces chemical alterations of the original compounds, thereby enabling their elimination. A second, complementary process is the active secretion to the bile duct from which the compound is further transported to the gastrointestinal tract. In pharmacology and medicine, plasma clearance is used to quantify the rate by which a compound is eliminated from the body [1]. Plasma clearance describes the overall detoxification capacity of the organism and summarizes individual clearance rates from all eliminating organs with the largest contribution coming from the kidney and the liver. While renal clearance can be measured by urinary secretion, a quantification of liver detoxification capacity is difficult since the different physiological functions cannot be assessed directly. In particular, the relative contributions of the different underlying physiological functions such as metabolization or biliary secretion cannot be adequately differentiated since the liver is frequently rather considered as a net systemic sink. While hepatic turnover can be indirectly quantified with drugs following a known pharmacokinetic profile, the local, time-resolved distribution of compounds within the whole organ can generally not be analyzed even with distinguished measurement techniques. This holds in particular for the first pass of drug perfusion in a liver directly after drug administration, when hepatic tissue is exposed to a novel xenobiotic for the first time.

Due to the pivotal role of the liver in drug pharmacokinetics and detoxification, several models quantifying hepatic clearance have been developed before [2]. These include tube models for representative hepatic sinusoids (single tubes, in parallel or in series) [3], dispersion liver models [4], fractal liver models [5], circulatory models [6], [7] including zonal models [8], [9], and distribution-based models describing statistical variations in transition times [10]. For a more detailed overview, we refer to [11].

Generally, PK models are well suited to investigate distribution and clearance of compounds in the body. In compartmental PK modeling, a limited number of rather generic compartments is usually used to simulate plasma concentration and drug clearance. Following a complementary approach, physiologically based pharmacokinetic (PBPK) models describe biological processes at a large level of detail based on prior physiological knowledge [12]. This involves amongst others organ volumes and blood flow rates, such that physiological mechanisms underlying absorption, distribution, metabolization and excretion of compounds can be explicitly described. While most approaches consider intravenous or oral application of therapeutic compounds, PK models describing further uptake routes such as inhalation have also been developed [13], [14]. Organs in PBPK models are divided in several subcompartments. So-called distribution models are used to describe the mass transfer between these subcompartments which are quantified based on physicochemical compound information such as lipophilicity or the molecular weight. Basic PBPK models can be extended to include enzyme-mediated metabolization or active transport across membranes [15].

Coming from the field of toxicology [16], PBPK models are nowadays routinely used in drug discovery and development [17]. They are for example applied in pediatric scaling [18], model-based risk assessment [19], as well as for multiscale modeling by integrating detailed models of intracellular signaling [20] or metabolic networks [21] into the cellular subcompartment, thus allowing for the analysis of cellular behavior within a whole-body context [22]. Notably, each organ in PBPK modeling is generally represented by few well-stirred subcompartments, thus allowing a quantitative description of drug pharmacokinetics once an equilibrium between the vascular system and the surrounding tissue has been reached. However, a spatially resolved description of drug perfusion in the whole organ covering particularly the first instants after drug administration is impossible due to the inherent well-stirred assumption.

**Figure 1:**
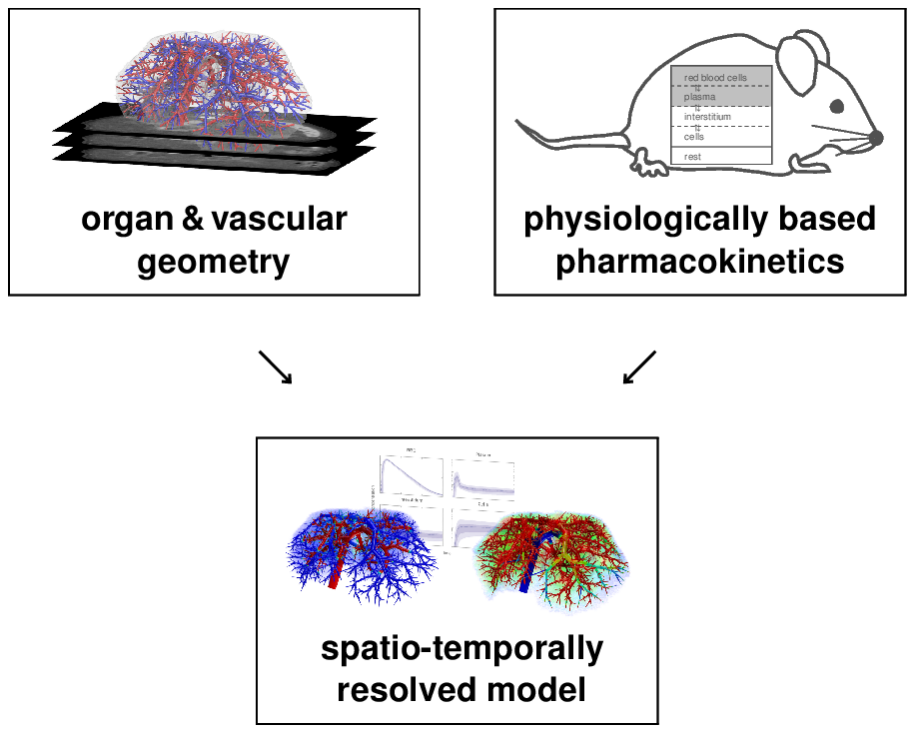
Conceptual model overview. Our spatially resolved model is based on mass balance equations from physiologically based pharmacokinetic modeling as well as organ and vascular geometry obtained by in vivo imaging. The combined model allows a detailed simulation of hepatic distribution and metabolization to accurately describe spatio-temporal effects underlying first-pass perfusion in the liver.

To mechanistically describe first pass perfusion, we here present a spatio-temporal model of drug distribution and metabolization in a mouse liver. The model represents liver lobes at the spatial length scale of lobuli such that physiological and molecular processes can be simulated in great detail. Our combined spatially resolved model (cf. overview in Figure 1) involves three main building blocks. These comprise physiological vascular structures, an organ-scale perfusion model describing blood flow and advection of compounds, and finally pharmacokinetic models translated to the spatially resolved tissue. Geometrically accurate models of murine hepatic vascular structures were obtained by using in vivo micro-CT imaging [23]. The mass balance within the tissue was quantified based on equations coming from PBPK modeling. Our combined model was inspired by [24], where a lobule-scale perfusion model in more physical detail and also allowing for deformation of the porous medium is introduced. A model for cardiac perfusion using very similar modeling techniques is presented in [25]. It considers multiple geometric scales, but no draining vascular system and no metabolization.

Our spatially resolved model covers several scales of biological organization displayed at varying levels of resolution. The scales range from the organ level to the cellular space where metabolization and molecular transport take place. The vascular systems form the scaffolds which links the hepatic in-flow to the sinusoidal space and thereby to the lobulus level. The model considers one supplying and one draining vascular system (denoted by SVS and DVS, respectively), with a homogenized hepatic space (denoted by HHS) in between as a tissue representation. The HHS includes in particular the sinusoids which are not explicitly resolved as vascular structures. In the latter, blood flow is represented by a fluid transport model [26]. Microcirculation and microanatomy [27] are only considered in effective form. While more detailed perfusion and metabolization models on the lobular scale [28], [29] or the tissue-level [2] have been developed before, the representation of the HHS as a porous medium [30] is sufficient for our needs.

Experimentally, the combined model corresponds to an isolated perfused liver [31], [32], since recirculation of blood is not considered. The resolution of the model allows calculating local concentration profiles within the tissue which can for example be used to address heterogeneous phenomena such as spatially varying distributions of lipid droplets in steatotic livers. Spatial heterogeneity of pharmacokinetic parameters such as metabolic capacity can be taken into account. Likewise local exposure profiles of toxic compounds can be simulated such that off-target effects of drug therapy can be analyzed at a high level of resolution. Applications of spatially resolved perfusion and metabolization modeling include optimized design of therapeutic treatment where spatio-temporal perfusion effects are of relevance, e.g. hypothermic machine perfusion [33] or islet cell transplantations [34]. Moreover, such models can be used for planning drug delivery for which spatially heterogeneous distribution is an important property and crucial for administration design itself. Two major examples are intrahepatic injection of chemotherapeutics or radionuclides (see e.g. [35]), in particular in combination with optimization of catheter placement [36], and targeted drug delivery [37] where drugs are injected in bound form and released at the desired location by mild hyperthermia. Likewise, our model may support data processing and interpretation in imaging or diagnostics. We expect the spatially resolved model presented here to be the foundation stone of further mechanistic models describing the spatial organization of the liver in an unprecedented level of physiological detail.

## Materials and Methods

### Ethics Statement

The animal experiment was reviewed and approved by the local authorities (NRW LANUV, permit number 10576G1) according to German animal protection laws.

### Constructing a Combined Model

In the methods to be presented, we follow a geometric view from coarse to fine, i.e. from (1) the body scale (providing organ input and output) via (2) the vascular structures on the organ scale (perfusion only) to (3) the tissue scale (perfusion and metabolization). Models for steatosis and 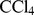-induced liver necrosis are subsequently presented to demonstrate how our spatially resolved simulations can be used for the analysis of pathological states influencing drug distribution in the liver. Finally, some aspects of computational resolution are addressed.

### Overall Model Structure

**Figure 2:**
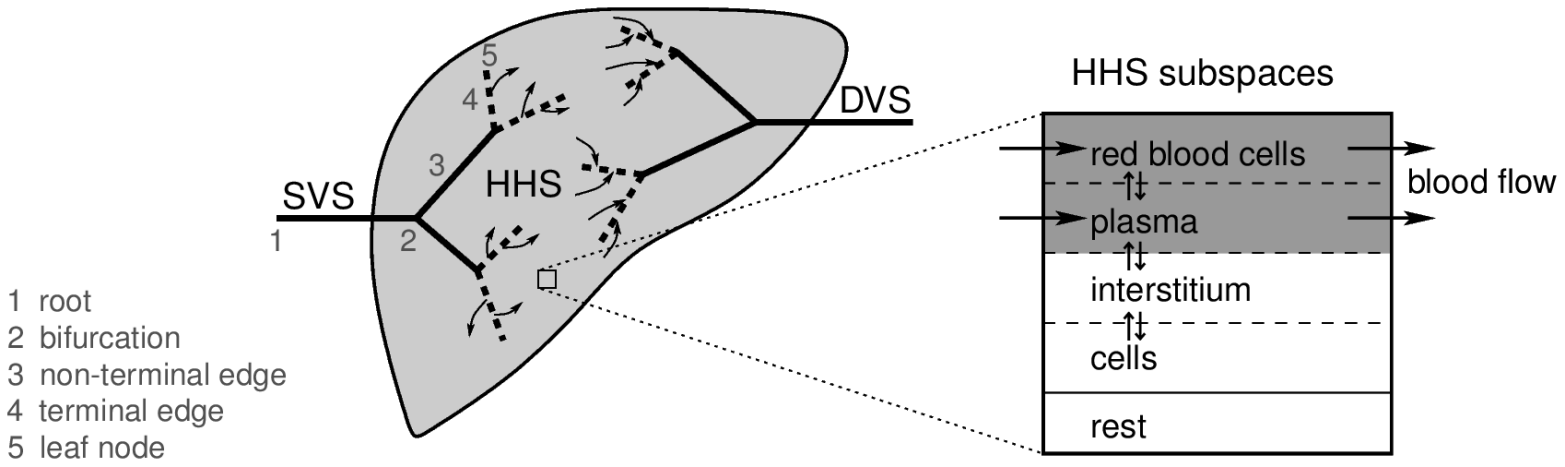
Conceptual two-dimensional sketch of our liver perfusion model. The homogenized hepatic space HHS is supplied and drained by the supplying vascular systems SVS and the draining vascular system DVS, respectively. The blood flow through the SVS summarizes the contributions of both supplying systems (hepatic artery and portal vein). The blood is hereafter transferred to the HHS along the terminal edges of the supplying vascular tree (dashed lines). After blood has passed through the HHS, flow into the draining vascular tree (hepatic vein) occurs again along its terminal edges (dashed lines). The HHS itself locally consists of several subspaces, the sinusoidal subspace (combining red blood cells and the plasma subspace), the interstitial subspace, the cellular subspace, and the remaining subspace. An actual 3D vascular geometry is shown in Figure 3. The SVS and DVS roots are connected to the rest of the body by the total blood flow in the liver. In the vascular structures, only 1D advection with given velocities per edge take place. In the HHS, 3D advection (according to a 3D flow velocity vector field) as well as exchange between the HHS subspaces and metabolization (according to PBPK model parameters) are considered simultaneously.

Our spatially resolved model distinguishes between the supplying vascular tree, the HHS and a draining vascular tree which are considered in series (Figure 2). For reasons of simplicity, the supplying vascular system comprises both the portal vein and the hepatic artery. Since an isolated perfused liver was considered here which explicitly excludes recirculation through the body, the respective contributions of the portal vein and the hepatic artery were not distinguished and only the total blood inflow was taken into account. The draining vascular system represents the hepatic vein. Bile ducts were not considered in our model, since their geometric structure could not be resolved experimentally.

In our combined model, the HHS is composed of several subspaces in analogy to the liver compartment in PBPK models. The latter is divided in four subcompartments, i.e. red blood cells (rbc), plasma (pls), interstitium (int), and liver cells (cell). Those four subcompartments are also considered as subspaces of the HHS, in addition a fifth remaining subspace (rest) is taken into account. This remaining subspace comprises all those parts inside the liver that are not considered for perfusion, metabolization and active transport, in particular the larger and explicitly resolved vascular structures and the bile ducts. The plasma subspace only refers to the blood plasma. For notational convenience, the sinusoidal subspace (sin) is defined as the combination of red blood cells and plasma subspace, thus representing a whole-blood compartment. The sinusoidal subspace is subject to advection, thereby reflecting blood flow through the tissue. The actual metabolization takes place only in the cellular subspace and is part of the PBPK equations that also model the exchange between the HHS subspaces. The vascular trees are resolved separately and considered for pure advection.

The volume fractions of the subspaces relative to the overall liver volume, also needed for the mass balance in the compartment models, are denoted by 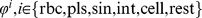. For our simulations in a mouse liver, we use the values
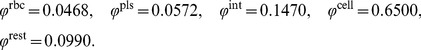
(1)Volume fractions were obtained by setting 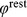 to cover the fraction of the vascular volume inside the segmented liver volume, both determined from the experimental image data as described below. The values for the other subspaces from [38] were then scaled accordingly by 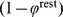 so that all five volume fractions sum up to 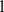. From Equation 1, we immediately obtain 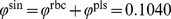.

For the perfusion part in our model, we address how molar concentrations 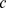 of compounds change due to advection through the vascular systems and the sinusoidal HHS subspace. The exchange with the remaining HHS subspaces and cellular metabolization are considered as a separate contribution to our combined model.

The body scale determines the total perfusion 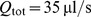 of the liver in mice [38]. The blood flow into and out of the root edges of the supplying and draining vascular system, respectively, is the connection of the HHS to the surrounding organism. More precisely, inflowing and outflowing molar concentrations of the compounds of interest are the main model input and output quantities.

### Organ Scale—Modeling the Vascular Systems

#### Obtaining physiological vascular geometries

**Figure 3:**
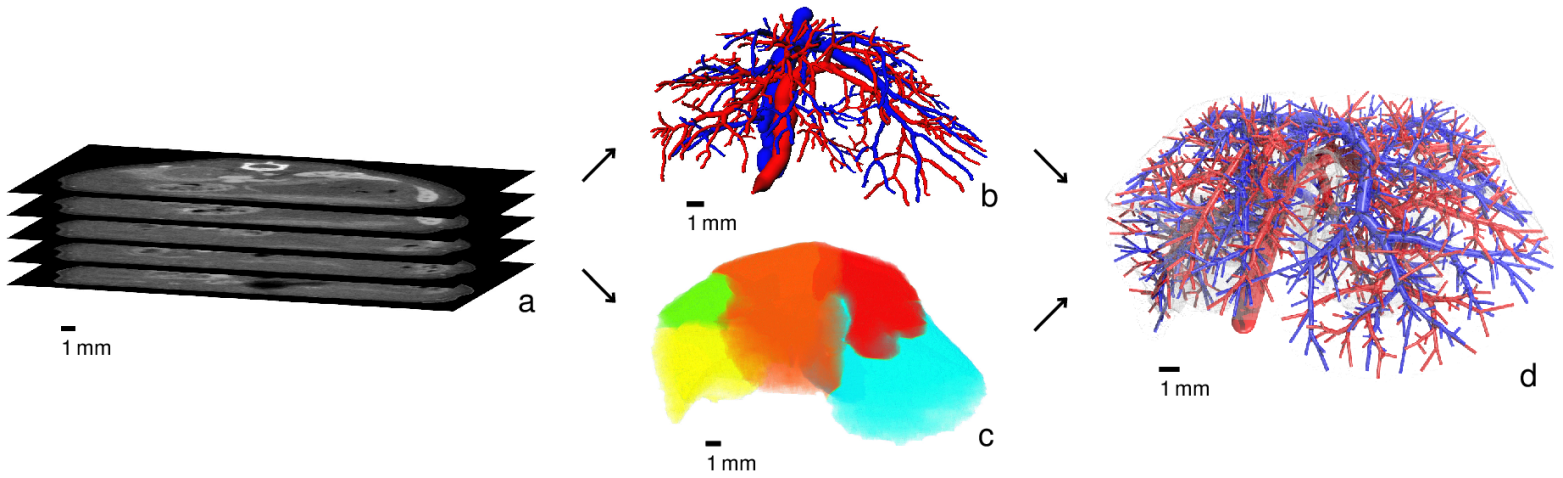
From in vivo scans to vascular geometry. Based on an in vivo micro-CT scan (*a*) of a mouse, vascular structures (*b*) in the liver are segmented and skeletonized. The supplying and draining vascular systems (SVS and DVS) are shown in red and blue, respectively. Furthermore, the liver is segmented and decomposed in lobes shown in different colors in (*c*). An algorithmic procedure is used to determine physiological vascular structures (*d*) with the desired level of detail, in our case 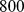 leaf nodes (end points) for each of the two trees are used.

The geometry of vascular systems and the organ shape used in our model was obtained from in vivo 3D images as illustrated in Figure 3.

To assess the vascular systems in vivo, a contrast enhanced micro-CT scan was acquired [23] for a nude mouse. The two tubes of the micro-CT (CT-Imaging, Erlangen, Germany) were operated at 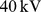/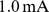 and 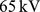/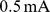. For each micro-CT sub-scan, 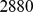 projections containing 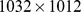 pixels, were acquired over 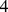 full gantry rotations with a duration of 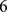 minutes per sub-scan. To cover the whole body, three sub-scans were acquired, each covering 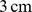 in the axial direction. Before scanning, the mouse received an intravenous injection (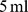 per kg body weight) of an iodine-based radiopaque blood pool contrast agent [39]. During imaging, the mouse was anesthetized using a 2% mixture of isoflurane/air. After scanning, a Feldkamp-type reconstruction was performed at isotropic voxel size 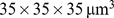 using a ring artifact reduction method (CT-Imaging, Erlangen, Germany), the image dataset was subsequently downsampled by a factor of 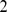 (isotropic).

From the CT image data, a liver segmentation (including decomposition in liver lobes) and a graph representation of the vascular structures (portal vein and hepatic vein) was determined using segmentation and skeletonization by a semi-automatic and clinically evaluated [40] procedure described in [41]. The graph was transformed into a strictly bifurcative tree with cylindrical edges (vascular pieces between two points, bifurcation to next bifurcation, or end point to next bifurcation, see Figure 2) of constant radius following the methods in [42]. The resulting liver has a volume of 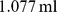. The obtained vascular graphs containing 443 and 299 leaf nodes (end points) for the portal and hepatic vein, respectively, are insufficiently detailed for robust simulations, so they need to be refined algorithmically.

For this purpose, an implementation [42] of a constrained constructive optimization method [43] for non-convex organ shapes was used. In summary, the algorithm determines a set of leaf nodes, each representing one lobule or a group of lobuli. It then starts with an initial tree, here obtained from an in vivo CT scan, and connects the additional leaf nodes by one. Each time, the algorithm introduces a new bifurcation which is optimal in the sense of minimal intravascular volume while providing constant blood supply for each leaf node. In finely resolved hepatic vascular trees of mice, there are many edges with radius less than 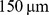, so it is particularly important to take the decrease of effective blood viscosity due to the Fhrus-Lindqvist effect [44] into account for the constructive algorithm. The methods of [42] were extended by more strictly avoiding vascular connections outside the organ. Note that this algorithm yields physiologically realistic vascular structures but is not meant to model the vascular development during organogenesis as studied e.g.in [45]. The algorithmic procedure is illustrated in Video S1.

#### Perfusion simulation in the vascular structures

We assume the same outflow from each leaf edge of the supplying vascular system and the same inflow to each leaf edge of the draining vascular system matching the assumptions in the constrained constructive optimization framework [42] used for vascular refinement. In particular, homogeneous perfusion is an assumption currently put into the model and no result.

In the vascular systems, we have essentially one-dimensional (1D) advection with velocities determined by flow splitting satisfying mass conservation and the underlying geometry, namely cross-section areas of edges. There is no diffusion term in the model, we can, however, not avoid artificial numerical diffusion [46] introduced by the discretization as discussed in Section 1 in the Text S1.

Advection of a molar concentration profile 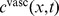 of a single compound through the vascular systems can be described by 1D advection as we assume a constant mean velocity 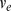 for each vascular edge 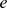. As there are no sources inside the vascular structures, this is described by the partial differential equation (PDE)
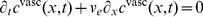
(2)with appropriate initial conditions, boundary conditions at inflows, or coupling to connected edges. In the combined model, two advection problems of this form per compound are considered, one for the plasma and one for the red blood cells (see Figure 2). These are independent of one another and the same flow velocity is assumed for both.

At branching points in the supplying vascular system, mass conservation implies a splitting of the blood volume, but the molar concentrations do not change. In contrast, at branching points in the draining vascular system we assume instant mixing of the molar concentrations. These are obtained as the average of the inflowing molar concentrations weighted with the respective volume flow fractions. Mass conservation in the 1D model is ensured by the flow velocities. For discretizing the advection problem in Equation 2 for vascular trees, we use a 1D Eulerian-Lagrangian Locally Adjoint Method (ELLAM) [47] scheme adapted to the case of branchings. ELLAM allow for flexibly integrating other phenomena than advection in the discretization and avoids numerical artifacts occurring in other discretization schemes for advection problems [48]. Details about the discretization are explained in Section 1 in Text S1, the implementation is an extension of own earlier work [49]. Alternative techniques for discretizing and simulating flow have been developed before, see e.g. [50].

The inflow in the root of the supplying vascular system from the body scale is given by a time-dependent molar concentration 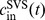. Outflow from terminal edges of the supplying vascular system is treated by applying an appropriate molar concentration sink term. Mass conservation is ensured by vascular sink terms being HHS source terms as discussed below.

In the draining vascular system, the flow of information is different. Terminal edges drain whatever molar concentration is present in their vicinity, and the molar concentration obtained at the root is given back to the body scale as a time-dependent molar concentration. For this purpose, the inflow at each leaf node is computed as the average molar concentration near the corresponding draining terminal edge. Again, mass conservation is ensured by a corresponding sink for the HHS, see below. The outflow from the root edge of the draining vascular system does not require special treatment in the advection simulation, it merely needs to be evaluated for each time step.

### Tissue Scale—Modeling the Homogenized Hepatic Space

The HHS is modeled as a porous medium representing the effective behavior of the whole liver volume, with the sinusoidal subspace being perfused according to Darcy's law [51]. The perfusion is split between the red blood cells and plasma subspaces (see Figure 2) proportional to their respective volumes.

Using appropriate flow source and sink terms in the HHS corresponding to the exchange with the vascular systems, flow velocities for 3D advection in the HHS are obtained. The advection of concentrations can then be calculated from the given flow velocities using appropriate discretizations described in this section. Technical details about the discretization and implementation in the model are presented in Section 2 in Text S1. Finally, the pointwise exchange in the spatially resolved model between different HHS subspaces and the actual metabolization are modeled by equations coming from PBPK modeling.

#### Obtaining flow velocities

While the flow velocities in the vascular systems are easily obtained from the flow splitting described above and the respective cross-section areas, this is more complicated in the HHS. Flow in the HHS is determined by radial outflow and inflow for the terminal edges (see Figure 2) of the SVS and DVS, respectively. This outflow and inflow is assumed to be constant along each edge and assumed to happen along the one-dimensional line segments being the center lines of the terminal edges where we define source and sink terms 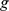 used in Equation 4.

The Darcy velocity vector field for the blood flow in the sinusoidal subspace of the HHS is obtained as
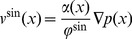
(3)where 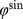 is the appropriate porosity of the HHS (sinusoidal volume fraction), 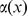 is the effective permeability defined as permeability divided by dynamic viscosity, and 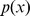 is obtained from
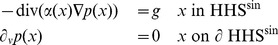
(4)where 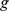 are the one-dimensional flow sources and sinks due to the terminal edges as discussed above. Rather than using the two quantities permeability and dynamic viscosity separately (as in [51] and commonly done in the literature), we here only need their ratio 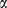.

For constant effective permeability (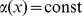), its absolute value in this setting does not matter for the velocities obtained, since 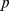 scales linearly in 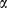 for given flow sources 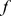, and the velocity 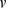 scales linearly in 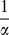 for given pressure 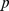. We hence assume the effective permeability to be 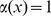. This means that the quantity 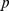 cannot be interpreted as the actual physical pressure present in the HHS, but merely as a relative pressure. However, 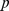 is just an internal quantity of the computation described above and only the resulting velocity 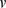 will be used later on.

Equation 4 was discretized using standard trilinear finite elements for computing the pressure 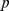, using the appropriate line integrals for computing the right hand side source terms. The hexahedral finite element grid for this purpose was chosen to be the voxel midpoints of the binary image segmentation of the liver as obtained from the image processing. A piecewise constant velocity profile 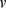 was obtained from the pressure 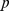 using difference quotients corresponding to the given grid when discretizing the gradient in Equation 3. Due to the discretization and the lower-dimensional sources, the resulting velocity field will not have exactly vanishing divergence. This needs to be taken into account when simulating advection in the next step.

#### Perfusion simulation in the Homogenized Hepatic Space

Using the velocity profile 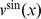 of blood in the sinusoidal subspace from Equation 3, we can now simulate the advection of molar concentrations 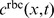 and 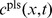 of compounds. For simplicity, we will restrict the presentation to a single compound. As 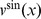 is constant in time, advection is described by the PDEs
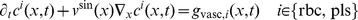
(5)where the 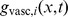 are lower-dimensional sources and sinks describing the inflow and outflow of compounds through the terminal edges of supplying and draining vascular system, respectively. Again, the velocity is the blood flow velocity and thus the same for any compound. In our simulations, constant initial conditions for Equation 5 were used. An explicit treatment of boundary conditions is not necessary since the velocity vanishes in normal direction to the liver boundary.

The molar concentration transfer from the supplying vascular system to the HHS is modeled as follows. In each terminal edge of the supplying vascular system, compounds are transported along the whole length to the end point and out of the edge. As we assume the cross-section area to decrease linearly to zero, this corresponds to a constant outflow of mass along the terminal edge. This mass outflow is used as a one-dimensional source term in the HHS, satisfying mass conservation. This models a flow to connected smaller vessels or sinusoids from the last resolved vascular edges.

Terminal edges of the draining vascular system are assigned an inflow value, in the same manner as for the root edge of the supplying vascular system. This inflow value is determined as an average molar concentration in a neighborhood of the terminal edge. Mass conservation is ensured by considering a corresponding one-dimensional source term with negative sign in the HHS. Note that this is only an approximation of an inflow from sinusoids or smaller vessels into the first resolved vascular edges.

In the supplying vascular system, we assume inflow concentrations to be such that an equilibrium between red blood cells and plasma concentrations has been obtained before the injected compound reaches our liver model.

The discretization of Equation 5 using a 3D ELLAM scheme is described in Section 2 in Text S1.

#### Metabolization simulation using Physiologically Based Pharmacokinetic Models

The exchange between the different HHS subspaces and the metabolization in the cellular HHS subspace are described by PBPK model equations. In the simulations performed, we ignore enzymatic formation of metabolic by-products, i.e. we consider the metabolization as a sink, and thus need one inflowing and one outflowing molar concentration only.

As mentioned above, PBPK models divide the liver in four subcompartments, i.e. red blood cells, plasma, interstitium, and cells. The PBPK models were written in terms of molar concentrations, so that a pointwise exchange 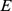 between the different subspaces and the contribution of metabolization 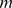 can be written in the form
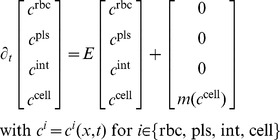
(6)with 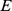 and 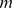 defined in Equations 7 and 8a/8b. We here omitted the dependency of concentrations on space and time to simplify notation. Note that perfusion and compound inflow are considered separate from Equation 6.

In all our PBPK models, only passive, gradient-driven exchange of compounds takes place. We thus write Equation 6 using a 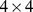 matrix to quantify exchange 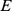 of compounds as
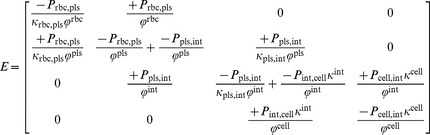
(7)where 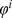 are the volume fractions from Equation 1; 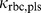, 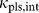, 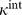, and 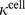 are dimensionless partition coefficients describing the equilibrium state of molar concentrations for which the exchange vanishes; and 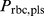, 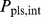, 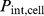, and 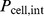 are the local effective permeabilities [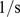] between the different subspaces of the HHS [12].

The actual metabolization 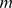 within the cellular subspace is captured by linear mass action or Michaelis–Menten kinetics [52]

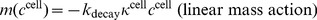
(8a)

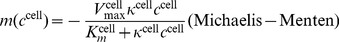
(8b)with a first order rate constant 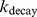 [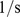] or Michaelis–Menten parameters 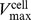 and 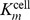.

Our PBPK models were built using the software tool PK-Sim (version 5.1; Bayer Technology Services GmbH, Leverkusen, Germany). The PBPK models generated in PK-Sim were exported and modified in MoBi (version 3.1; Bayer Technology Services GmbH). All PBPK models consider the pharmacokinetic characteristics of absorption, metabolization, and excretion of the simulated drug. Physiological parameters describing basic model structure such as organ volumes, blood flow rates, or surface permeabilities are provided by the software tool [12], [53]. Mass transfer in PBPK models is described by so-called distribution models which are parametrized based upon the physicochemical properties of the compound under investigation. Notably, all physiological parameters are either explicitly provided in the PBPK software, e.g. organ volumes or blood perfusion rates, or they can be calculated by means of the underlying distribution model. In the latter case, physicochemical properties of the substance such as lipophilicity (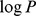) or molecular weight (MW) are used to quantify corresponding model parameters. The overall number of independent parameters in PBPK models is hence low (usually in the order of 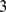 to 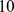).

For each of the three exemplary compounds considered here, PBPK models were developed, i.e. the respective model parameters involving local effective permeabilities or partition coefficients were adjusted with respect to plasma concentration data. Metabolization parameters (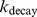 or 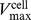 and 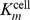) were obtained by comparing simulation results of a whole-body PBPK model to experimentally measured plasma concentrations of the respective compounds. In contrast, permeabilities and partition coefficients are derived from physicochemical properties of the compounds. In order to quantify the model quality, we computed concordance correlation coefficients [54] for experimentally measured concentrations and simulated concentrations at the same time points. Once the PBPK models were established and found to describe the experimental data with sufficient accuracy, parameters describing the mass transfer in between the four subcompartments were used to quantify the corresponding processes in the spatially resolved model of the isolated liver, see Table 1 for the resulting parameters.

**Table 1:** PBPK parameters for the compounds considered.

	CFDA SE	Midazolam	Spiramycin
MW [ 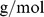 ]	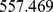	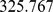	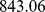
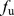 [–]	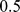 *	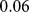 [91] §	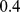 [92]
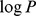 [–]	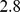 †	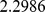 [91] §	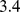 [92]
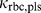 [–]	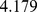 ‡	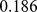 ‡	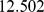 ‡ ∥
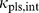 [–]	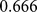 ‡	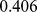 ‡	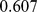 ‡
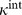 [–]	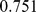 ‡	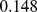 ‡	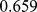 ‡
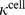 [–]	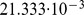 ‡	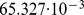 ‡	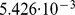 ‡
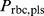 [ 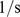 ]	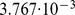 ‡	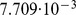 ‡	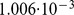 ‡ ∥
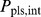 [ 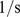 ]	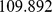 ‡	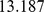 ‡	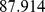 ‡
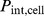 [ 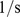 ]	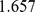 ‡	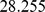 ‡	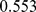 ‡ **
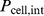 [ 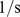 ]	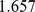 ‡	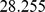 ‡	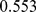 ‡ **
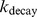 [ 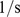 ]	n/a	n/a	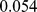 ¶
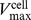 [ 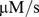 ]	n/a	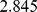 ¶	n/a
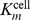 [ 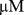 ]	n/a	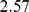 ¶	n/a

The table lists the PBPK model parameters for CFDA SE, midazolam, and spiramycin used in our simulations, including literature sources. Molecular weight MW, fraction unbound 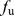 and lipophilicity 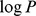 are used to calculate the partition coefficients 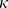 and permeabilities 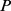 used in the PBPK models. The physicochemical compound parameters were fine-tuned with respect to initial literature values by comparing PBPK simulation results to experimental PK data. Likewise, parameters 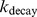, 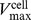, and 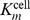 quantifying turnover in the metabolization terms were fitted to experimental PK data during model establishment.

* estimated.

† chemformatic prediction.

‡ computed from the PBPK distribution model [38] based on MW, 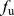, and 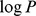.

§ fine-tuned from literature values during model establishment with respect to experimental PK data.

¶ fitted during model establishment with respect to experimental PK data.

∥ unused in the experimental setting [32] without RBC.

** adjusted later for the experimental setting [32].

#### Combined perfusion and metabolization model

Exchange of compounds between the different subspaces of the HHS (see Figure 2) and cellular metabolization is modeled as the PBPK term from Equation 6 combined with the advection term from Equation 5. More precisely, advection applies to 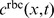 and 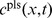, the PBPK term additionally involves 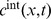, and 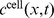. Again omitting the dependency of the parameters and concentrations on space and time to simplify notation, we can write the combined advection-PBPK problem as an extension of Equation 6,
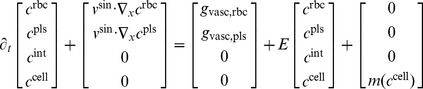
(9)with 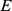 and 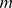 as explained in Equations 7 and 8a/8b, respectively.

Let us sum up the dependency of parameters on space and time. For our purposes, the velocity 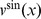 in Equation 9 only depends on space, the source terms 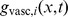 depend on space and time. Effective permeabilities and partition coefficients as well as the metabolization parameters are constant in space and time except for 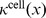 in a spatially heterogeneous steatosis model described below.

Exchange and metabolization could be integrated in the ELLAM scheme used for discretizing the HHS perfusion, even if they are nonlinear [55]. Implementing such a combined ELLAM scheme, however, is quite complex and requires a timestep appropriate for both phenomena. Instead, we consider the two phenomena separately and use alternating time steps (see below for the choice of time step sizes). The advection part is discretized in space and time by a 3D ELLAM scheme as described above and in Section 2 in Text S1. For the PBPK part as in Equation 6, time stepping is necessary for each grid node, and we use standard Runge-Kutta-Fehlberg 4th/5th order (RKF45) time stepping [56] which automatically adapts the internal time step size. This amounts to solving the advection equation 5 involving all of the grid points in each step and the PBPK part of Equation 9 separately for each grid point.

The simulations, including determining the 3D velocity vector field in the HHS were implemented in custom C++ code using the QuocMesh software library (version 1.3; AG Rumpf, Institute for Numerical Simulation, University of Bonn, Germany).

### Steatosis and Necrosis as Examples for Heterogeneous Pathophysiological States

The final spatially resolved model can also be used for analysis of pathophysiological states of the liver which have not been taken into account during model establishment itself. We here considered the case of steatosis leading to changes in intracellular lipid content as well as carbon tetrachloride (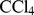)-induced liver necrosis affecting the spatial composition of the organ. Describing pathophysiological changes in spatially heterogeneous patterns is a key strength of our approach. A comparison of the simulation results with experimental data allows to evaluate model validity, thereby providing targeted suggestions for model extensions and modifications.

Steatosis is a common liver disease often caused by obesity or alcohol abuse [57]. It is characterized by lipid accumulations in the cellular subspace [58], the influence of which can be structurally represented in the model. We here analyzed to what extent steatosis affects hepatic clearance following changes in intrahepatic drug distribution. For our simulations we consider data reported from rats in [59, Table 8], namely steatosis extents of about 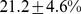 and 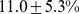 (mean 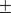 SD) in the left lateral and median liver lobe, respectively, obtained after two weeks of a specific diet. We proceed assuming that similar steatosis patterns can also exist in mice.

**Figure 4:**
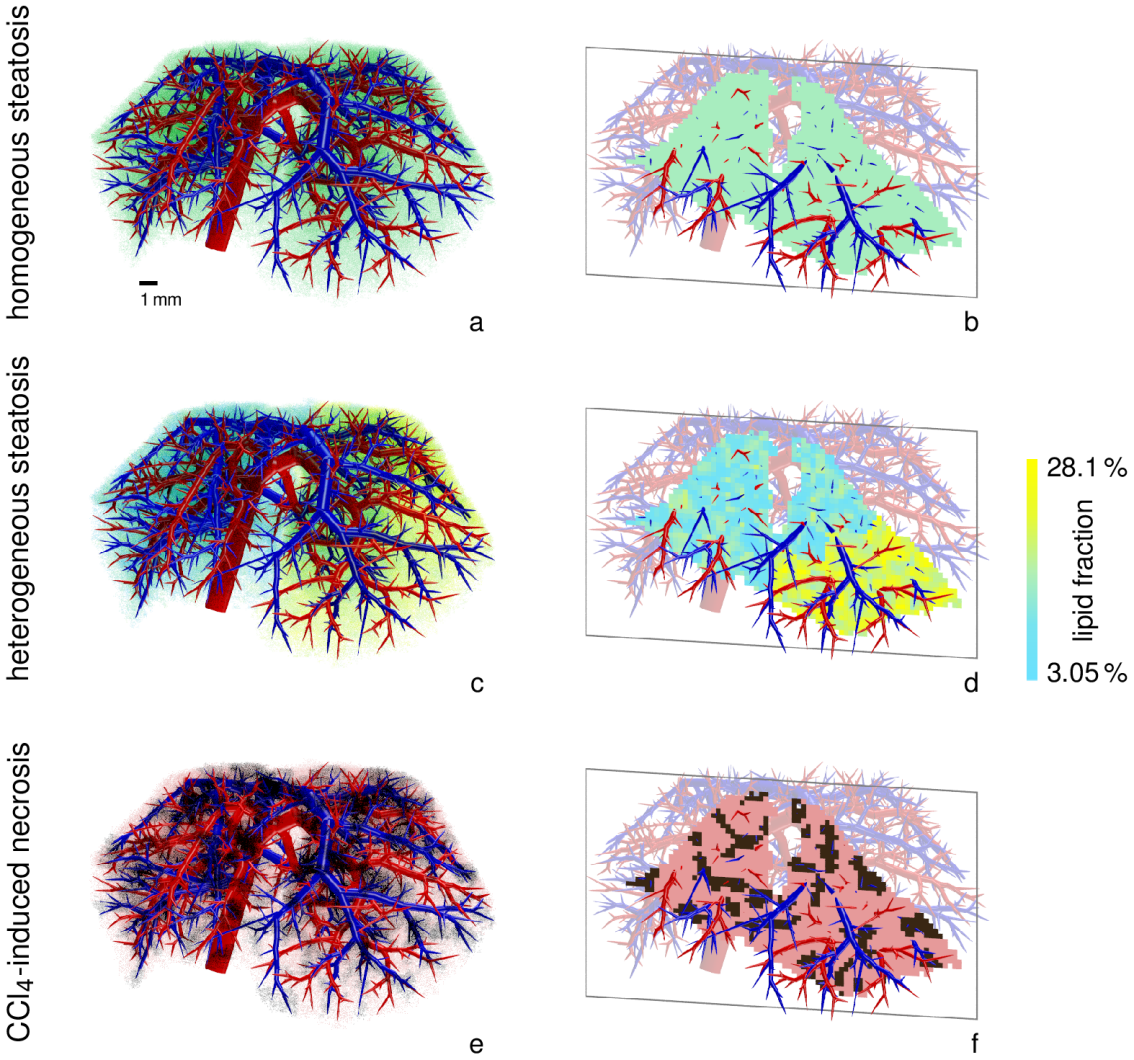
Visualization of pathophysiological states (steatosis, 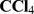-induced necrosis) in the spatially resolved liver model. The images show the distribution of lipid in our steatotic model with homogeneous lipid accumulation throughout the whole liver (*a, b*) and different heterogeneous distributions (*c, d*) in the left lateral lobe and the remaining lobes. The average lipid accumulation over the whole liver is the same in both steatosis cases. The lipid accumulation is assumed to change the distribution and metabolization behavior according to Equation 9. The liver volume affected by 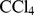-induced necrosis is shown in dark at the bottom (*e, f*). In each case, a volume rendering (*a, c, e*) and one coronal slice through the model liver (*b, d, f*) is shown along with the vascular structures.

Let 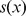 be the ratio of lipid accumulation per liver volume at position 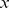, corresponding to the steatosis percentages in [59, Table 8]. Using the lobe decomposition of our mouse liver dataset (cf. Figure 3), we consider two states of steatosis. First, we use a homogeneous lipid accumulation 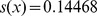 throughout the liver. This value is obtained as the average 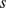 for 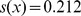 in the left lateral lobe and 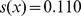 in the remaining lobes as the left lateral lobe in our case has 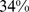 of the total volume. Second, we assign a pseudo-randomly varying value 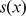 uniformly distributed in 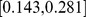 (left lateral lobe) and 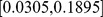 (remaining lobes) to obtain a spatially heterogeneous steatosis pattern. To avoid unphysiologically large local variations, we generated random numbers [60] on a grid four times coarser than the computational resolution and interpolated multilinearly at the nodes actually used. The two steatotic cases are visualized in Figure 4. In this setting, 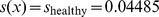 corresponds to the healthy state [12].

We quantify the impact of steatosis (an increased intracellular volume fraction 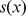 of lipids) by changing in cellular partition coefficient 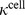 in the PBPK model. Any other effects of steatosis are explicitly omitted here for the sake of simplicity of this proof of concept for a spatial heterogeneity. The cellular partition coefficient is calculated according to the formula [12]

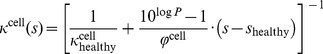
(10)with a constant 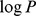 specific for the respective compound and 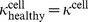 (Table 1).

The values 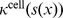 are substituted in Equations 7 and 8a/8b. As 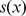 varies spatially, also 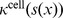 and thus intracompartemental exchange and metabolization vary accordingly. This is in contrast to commonly used PBPK models that, due to their compartmental organ representation, cannot distinguish between the homogeneous and heterogeneous case as they only use one constant value of 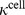.

As a second example for pathophysiological changes in the liver, we consider the case of 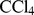-induced hepatic injury. Administration of 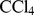 in animal models is a frequently used experimental protocol to investigate the processes underlying toxic liver damage [61]. Inducing hepatic injury by 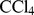 leads to necrotic cell death in the pericentral zone, similar to acetaminophen overdoses [62]. We analyzed the impact of pericentral necrosis on hepatic metabolization capacity. In our spatially resolved model, 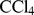-induced necrosis was represented by replacing the cellular space by interstitial space in the necrotic volume. The latter was set to be the 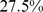 of the liver volume closest to the DVS terminal edges (see Figure 4), where the percentage is based on the area analysis in [63, Table 1], observed one day after 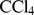 administration.

### Computational Resolution

Our basis for choosing computational resolutions is the actual size of lobuli in mice. From a cross-section area of 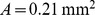, a radius of 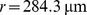 (assuming a regular hexagonal shape), both from [63, Table 1], and assuming the same elongation (length divided by diameter) of 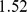 as for human lobuli [64, Chapter 2.5], a mouse lobulus has a volume of approximately 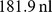, the total liver volume of 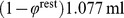 thus corresponds to 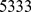 lobuli. By definition, a lobule is the volume drained by one terminal edge of the hepatic vein, so a fully resolved vascular tree has approximately that many leaf nodes.

#### Computational resolution for the Homogenized Hepatic Space

The grid spacing for discretizing the HHS was chosen to be 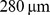, or one eighth of the image resolution of the CT image data, or approximately the lobulus radius. This choice leads to 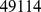 grid points inside the liver used in our simulations. Furthermore, anisotropy due to the internal arrangement of lobuli does not need to be taken into account at this resolution. Investigating the influence of discretizations other than hexahedral meshes considered here and their computational resolution requires a more elaborate investigation and is beyond the scope of this study.

#### Level of detail in the vascular tree

For the simulations presented later, 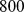 leaf nodes in both the supplying and draining vascular system were chosen as a trade-off between model accuracy (a fully resolved vascular system would have 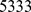 leaf nodes) and computational efficiency (i.e. using a small number of leaf nodes). Less than 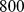 leaf nodes in the vascular trees was observed in Section 3 in Text S1 to lead to notable changes in the results while more details only lead to increasing computational costs. The vascular systems used for our simulations are visualized in Figure 3d and Video S2.

Since we do not fully resolve the vascular trees down to the lobular scale, the flow distance between the two vascular trees does not coincide with the real size of liver lobuli. Zonation effects [65], e.g. as observed in the simulations for the steatotic cases below, are qualitatively correct nonetheless. This is because the time available for metabolization is constant regardless of the vascular resolution, as we verify in Table 1 in Text S1, and because we do not consider individual cells (hepatocytes or other) and in particular do not see their length scale in our model. Consequently, also the overall clearance is represented correctly even though the vascular trees are not fully resolved.

**Figure 5:**
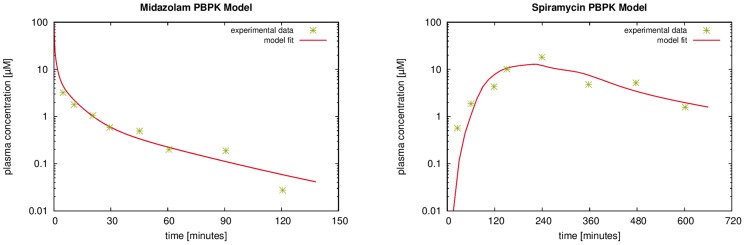
PBPK model establishment and parameter identification. Pharmacokinetic simulations of an intravenous dose of midazolam of 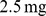 per kg body weight *(left)* and an oral dose of spiramycin of 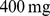 per kg body weight *(right)* are shown. The PBPK simulations (red lines) were compared to experimental data (green asterisks) for midazolam [66] and spiramycin [67] in mice.

To avoid excessively small time steps in the vascular advection simulation due to very short edges, a minimum edge length of 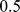 times the computational resolution in the HHS is enforced. Shorter terminal edges are pruned from the tree, shorter non-terminal edges are contracted to multifurcations. There is no further coupling between the discretization grids of the HHS and vascular structures as illustrated in Figure 5 in Text S1.

#### Choice of the time step

**Figure 6:**
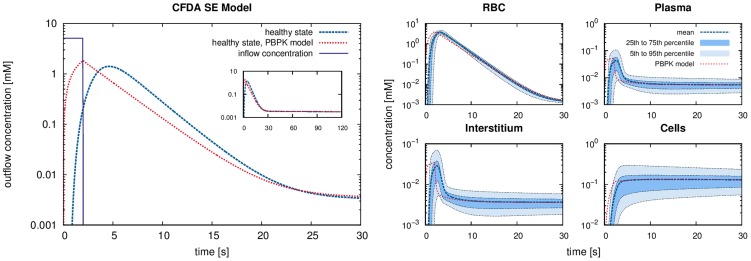
Results of the single pass perfusion of CFDA SE (outflow concentrations). For perfusion by CFDA SE, the large plot *(left)* shows the outflowing CFDA SE concentration in the healthy state of the isolated mouse liver model and the two steatotic states for a CFDA SE inflow during 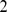 seconds. For comparison, results for a PBPK simulation are shown as well. The four small plots *(right)* show the mean CFDA SE concentrations in the four subspaces of the homogenized hepatic space as well as the ranges between 5th/95th and 25th/75th percentiles, respectively, to illustrate the ranges of the concentrations in the spatially resolved model. The PBPK simulation results, shown for comparison, in contrast yield one value for each compartment at any given time point, representing only mean values.

A fixed time step 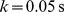 was chosen for the overall simulation (unless specified otherwise), and we alternatingly compute (1) advection time steps for the two vascular systems, (2) advection time steps in the HHS, and (3) metabolization time steps in the HHS. For (1), the time sub-step is chosen to be an integer fraction of 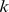 such that the condition in Equation 1 in Text S1 is satisfied. Similarly, a 3D analog as discussed in Section 2 in Text S1 needs to be satisfied for (2). As for (3), the RKF45 time stepping automatically and adaptively chooses appropriate sub-steps. The relation between the different time steps is illustrated in Figure 6 in Text S1.

## Results

To apply our model to pharmacological scenarios, we considered the distribution of three exemplary compounds covering typical aspects of drug distribution and metabolization: (1) the tracer carboxyfluorescein diacetate succinimidyl ester (CFDA SE), (2) the sedative midazolam, and (3) the antibiotic spiramycin. CFDA SE is a dye used to track proliferation in animal cells and is used here as a first proof of principle to describe the general behavior of our spatially resolved model only involving passive mass transfer. The model for CFDA SE was exemplarily used to investigate the computational performance and the influence of the level of detail in the vascular trees with regard to the number of leaf nodes (see Section 3 in Text S1). Also, we used the CFDA SE model to verify that the overall mass balance is satisfied in the combined model.

The PBPK model for midazolam was based on experimental PK data in mice [66] and considers both passive diffusion to the cellular subspace and consecutive hepatic metabolization by CYP3A. It thus extends the pure distribution model for CFDA SE by enzyme-catalyzed intracellular metabolization. Once established, the PBPK model of midazolam was used to quantify mass transfer and metabolization in the spatially resolved model. For spiramycin, we followed a similar approach by first establishing a murine PBPK model which is in agreement with experimental PK data [67], see Figure 5. Parameters used in our simulations are given in Table 1. The physicochemical properties of the three compounds together with the kinetic parameters quantifying metabolization are sufficient to parametrize the overall model structure of each of the PBPK models. All remaining parameters are either directly provided by the PBPK software such as organ volumes or they are calculated from the underlying distribution models based on the physicochemistry of the compounds.

We then used the PBPK model to parametrize the spatially resolved model. A comparison of the outflow concentrations of the spatially resolved model with experimental data from an isolated perfused liver [32] shows a good agreement with the experimental results. For all three compounds, we compared simulation results for the healthy reference state to homogeneous and heterogeneous steatotic states.

### CFDA SE—Distribution of a Tracer

As a first application example without metabolization, we considered the distribution of the tracer CFDA SE within the liver. Since adequate pharmacokinetic data for CFDA SE were not available for mice, a PBPK model could not be validated in detail. Instead, only the basic physicochemical parameters (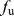 and 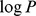) were estimated and subsequently used to calculate the parameters quantifying passive mass transfer in the PBPK model (Table 1). The pharmacokinetic behavior of CFDA SE was described by passive exchange as given in Equation 7. We considered an intravenous dose of 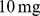 per kg body mass [68] corresponding to an inflowing concentration of 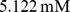 for a duration of 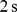 for a 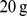 mouse. Note that the concentration of the compound in the inflowing blood encompasses the corresponding equilibrium concentrations in the red blood cells and the plasma, respectively. The model for CFDA SE was in particular used as a proof of concept for the general performance of the spatially resolved model. We could show with this model that overall mass conservation is satisfied, see Table 1 in Text S1. Since metabolization of CFDA SE was not considered here, concentrations of CFDA SE in the in- and the outflow alone could be used for this essential step in model validation.

The outflow curves for the spatially resolved model (Figure 6) show two effects, a temporal delay and a more smeared-out form of the peak from the spatially resolved simulation compared to the PBPK compartment simulation. The reasons for these observations become clearer when considering the temporal development of the concentrations in the four hepatic subspaces. The spatially resolved model no longer considers mean concentrations in well-stirred compartments but rather calculates heterogeneous distributions of these compounds. Likewise, the transition times needed to flow from the supplying to the draining vascular geometry are heterogeneous due to the different routes taken.

**Figure 7:**
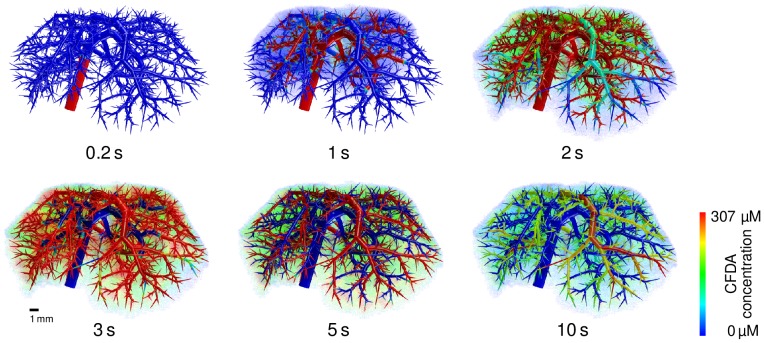
Results of the spatio-temporal perfusion simulations of CFDA SE in the liver. The volume renderings show the distribution of CFDA SE in the mouse liver for the healthy state at different time points, showing the first pass of perfusion (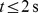), the distribution phase (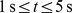) and the wash out (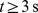).

We next visualized the total CFDA SE concentration in the HHS (Figure 7) obtained as the weighted average of the concentrations in the different subspaces,
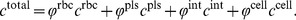
(11)Note that this is the quantity one observes in general for CT or MRI contrast agents by 3D imaging. In Figure 7 and in a Video S3, the different phases of the first pass of drug perfusion and distribution are shown. Also, the subsequent wash-out of the compound can be observed once the incoming pulse has passed through the liver. Notably, our spatially resolved model describes drug passage as a continuous process which may be used to complement experimental image data at discrete time points.

**Figure 8:**
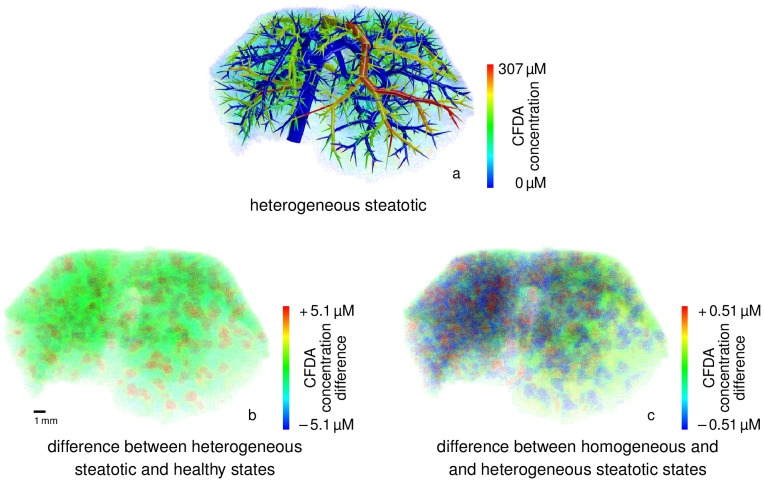
Influence of spatially heterogeneous lipid distributions on CFDA SE concentrations in steatosis. A comparison (*b*) of the CFDA SE concentrations at 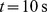 in the heterogeneous steatotic state (*a*) to the healthy state of the isolated mouse liver (see Figure 7) shows higher concentrations of the lipophilic tracer throughout the steatotic liver model. The difference (*c*) between the heterogeneous and homogeneous steatotic states exhibits higher CFDA SE concentrations (red spots) outside the left lateral lobe with higher lipid accumulation in the homogeneous case, see Figure 4. Notice that the color scales are different. This clearly shows that spatial resolution is indispensable for accurate modeling. For a clearer visualization of the concentration differences in the HHS volume, we omitted the vascular structures in the volume renderings (*b* and *c*).

Finally, we simulated steatotic cases where lipid accumulation in the cellular space of the liver influences the distribution behavior of compounds. In particular, we considered whether our spatially resolved simulations may be useful to support diagnostics and imaging. Concentration changes of CFDA SE due to spatially homogeneous and spatially heterogeneous states of steatosis are shown in Figure 8.

### Midazolam—Distribution and Metabolization of a Drug

As a pharmacokinetic application including intracellular metabolization, we next considered the distribution and metabolization of the sedative midazolam. For model establishment and parameter identification, we used previously published PK data [66] for mice obtaining an intravenous dose of 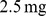 per kg body weight. Metabolization of midazolam by CYP3A was quantified by using gene expression data as a proxy for tissue-specific protein abundance within a whole-body context [15]. This also involves a specific quantification of the hepatic metabolization capacity which is an essential prerequisite for the consecutive parametrization of mass transfer in the HHS. The PBPK model of midazolam was pre-parametrized with the physicochemical compound parameters molecular weight, fraction unbound and lipophilicity. Subsequently, the compound parameters as well as the metabolization parameters were fine-tuned with respect to the experimental PK data [66] (Table 1). The simulated plasma time curves obtained with the thus established PBPK model are in good agreement with the experimental PK data in mice (Figure 5). For the midazolam PBPK model in Figure 5, a concordance correlation coefficient 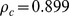 was found, see also Figure 3 in Text S1.

We next used the model parameters identified in the midazolam PBPK model for the spatially resolved model. As before, mass transfer of midazolam within the liver was described by passive exchange between the sinusoidal and interstitial subspace as well as the interstitial and cellular subspace as given in Equation 7. In addition, a nonlinear cellular metabolization according to Equation 8b was considered in this model. Values for the parameters in the equations are listed in Table 1. We considered a dose of 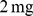 per kg body mass, corresponding to an inflowing concentration of 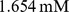 for a duration of 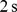.

**Figure 9:**
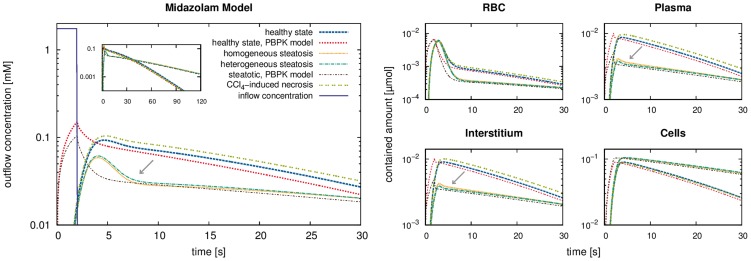
Simulations with a spatially resolved model for midazolam. The large plot *(left)* shows the outflowing midazolam concentrations for the healthy state and the pathological states for a midazolam inflow during 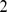 seconds. For comparison, results for simulations with a PBPK model are shown as well. The four smaller plots *(right)* show the total amounts contained in the subspaces of the liver, using the same lines and colors. Here, a difference between healthy and pathological states can be observed. In case of 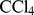-induced necrosis, higher outflow concentrations are predicted whereas they are lower in the steatotic cases. In particular, the outflow concentration as well as the amounts contained in the plasma and the interstitium also show a difference of up to 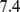, 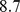, and 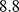 percent, respectively, between the homogeneous and heterogeneous steatotic states (marked by arrows).

Outflow concentration time curves from the draining vascular system for the healthy state are shown in Figure 9. The total molar amounts (concentrations integrated over the whole liver) of compounds contained in the red blood cells, plasma, interstitial and cellular subspaces are plotted in Figure 9. In the simulations, we again observe a delayed and more smeared-out peak in the spatially resolved model. After 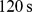 simulated time, our model predicts a metabolization of approximately 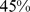 of the injected midazolam (healthy state), the rest having flown out from the model or still being present in the HHS and vascular systems. For midazolam metabolization, we also considered steatosis and 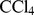-induced liver necrosis. In the homogeneous and heterogeneous steatotic state, an increase of the metabolization compared to the healthy state by 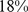 and 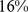, respectively, can be observed, again after 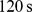 simulated time. For liver necrosis following 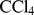 intoxication [61] our simulation predicts a decrease of 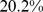 of the metabolized midazolam amount after 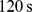.

### Spiramycin—Comparison to Experimental Data from an Isolated Perfused Liver

Finally we considered a model for the antibiotic spiramycin for which experimental data for an isolated liver were available in the literature [32]. For model establishment and parameter identification, we again used previously published PK data [67] for mice obtaining an oral dose of 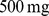 per kg body weight of spiramycin. Intravenous PK data are generally necessary for PBPK model development in order to identify systemic clearance capacity and distribution behavior without overlaying processes in the gastro-intestinal tract during oral absorption. Since intravenous PK data, however, were not available for mice, intravenous monkey PK data [69] were used for establishment of the fundamental model structure (Figure 1 in Text S1). We considered a linear metabolization term and pre-parametrized the distribution model with the physicochemical compound parameters (MW, 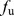, 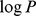). Based on the structure of the intravenous PBPK model, we then established a model for oral administration of spiramycin in mice [32]. Subsequently the model parameters were adjusted with respect to the experimental data [67] (Table 1). As before for midazolam, the spiramycin PBPK model provides a quantitative description of hepatic clearance capacity. The simulation time curves with the mouse PBPK model for intravenous spiramycin administration are in good agreement with the experimental plasma concentrations (Figure 5). For the PBPK model for spiramycin, we obtained a concordance correlation coefficient 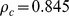, see also Figure 3 in Text S1.

Based on the validated mouse PBPK model for spiramycin we parametrized the spatially resolved model which is structurally identical to that of midazolam, except for the (now linear) metabolization kinetics. The spatially resolved model was then used to simulate experimental data for administration of spiramycin in an isolated liver [32]. The model structure of our spatially resolved model corresponds entirely to the experimental setup of the ex vivo assay, the availability of such highly specific data provided the opportunity to further validate our model. In the experiments [32], perfusion was performed using a buffer not containing red blood cells. The volume fractions from Equation 1 were hence changed to 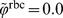 and 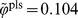. Moreover, a total perfusion of 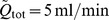 was used, which changes the flow velocities in our model and requires using a smaller time step (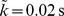). Passive exchange between plasma, interstitial, and cellular subspaces was again modeled as in Equation 7, mass transfer involving red blood cells, however, was set to zero to take into account the specific experimental setup [32]. Due to the unphysiologically high flow rate, the local effective permeability parameters between interstitial and cellular space were adapted to 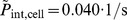 and 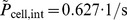. An inflowing spiramycin concentration of 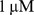 for a duration of 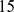 minutes was used as inflow condition reproducing the inflowing concentration profile in the experimental setup [32].

**Figure 10:**
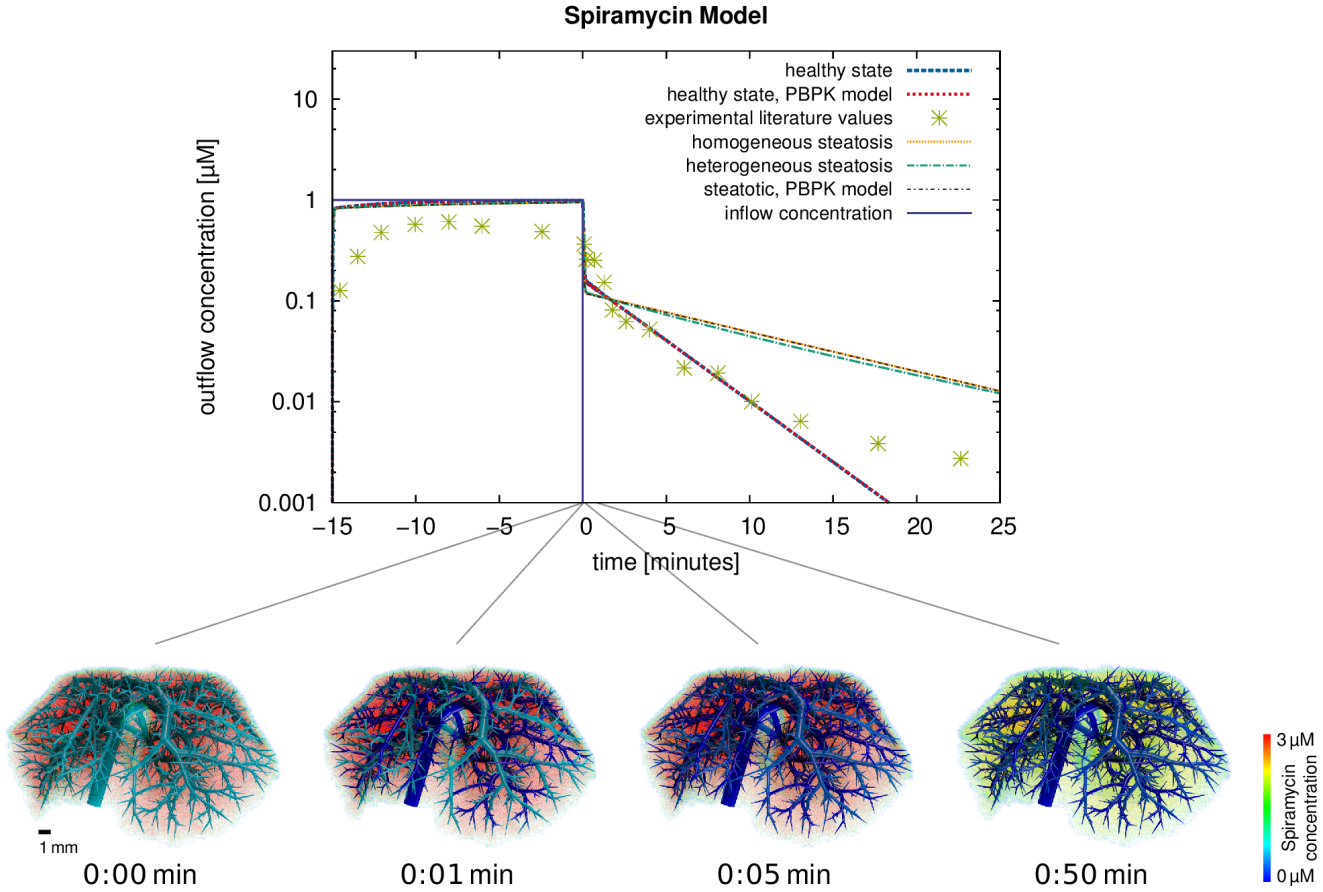
Results for the metabolization of spiramycin and comparison to experimental data from an isolated perfused liver. The plot shows the outflow rates of spiramycin from our single pass perfusion model for a spiramycin inflow during 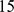 minutes compared to experimental data from an isolated perfused liver [32]. While the experimental values were measured in a healthy liver, we also show simulation results for the steatotic states. The volume renderings show the total spiramycin concentration for four time points after the end of the inflow (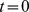 minutes).

For a comparison to the experimental data reported in Figure 2 (wild-type) in [32], the outflowing rate of spiramycin was computed and plotted in Figure 10, again for the healthy state and the two steatotic states described above. Comparing experimental outflow concentrations and those simulated using the spatio-temporal model for the healthy reference case, a concordance correlation coefficient 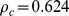 is obtained. Complementarily, volume renderings were generated at different time points after the end of the inflow for 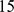 minutes (Figure 10) and show the spatial distribution of the spiramycin concentration immediately. This comparison illustrates very nicely how our spatially resolved model can be used to relate macroscopic observations in the plasma to distribution processes at the tissue scale.

## Discussion

### Simulation Results

We here present a spatially resolved model which describes the perfusion, distribution, and metabolization of compounds within the liver. The model structure is based on mass transfer equations obtained from PBPK modeling and vascular structures generated from micro-CT imaging. Our model excludes in particular any recirculation through the body such that metabolization and distribution of compounds can be considered without any overlaying effects. After the end of the initial administrations, a bi-phasic behavior can be observed which is initially governed by the distribution within the tissue and a slow release afterwards. Note that wash-out after the end of injection is additionally determined by advection in the blood flow.

Comparing outflowing concentrations from our spatially resolved simulations to those from PBPK compartment models showed a temporal delay, both for CFDA SE and midazolam (Figures 6 and 9). This is because the compound now needs to pass sequentially through the supplying vascular system, the homogenized hepatic space and the draining vascular system. Different paths through the liver model require different transit times, hence the peaks are more smeared-out in the spatially resolved simulations. This is further emphasized by the temporal development of the concentrations in the four hepatic subspaces for the CFDA SE simulations (Figure 6). The spatially resolved model no longer considers mean concentrations in well-stirred compartments but rather calculates heterogeneous distributions of the concentrations. Likewise, the transition times needed for the compounds to flow from the supplying to the draining vascular systems are heterogeneous due to the different routes taken. This shows the general performance of the spatially resolved model where mass flows follows the physiological architecture of hepatic tissue governed both by vascular geometry and the composition of the connecting hepatic tissue. While this temporal delay only plays a role during first pass perfusion or similar sudden incidents, results from the spatially resolved model can nevertheless be used to revise PBPK model parameters by comparison with targeted experimental data [32].

Previous approaches already described macroscopic effects such as transit time distribution [10], [11], this can also be reproduced using our model. In addition, our approach provides a mechanistic interpretation and visualization of the underlying processes. Our model allows for example a physiology-based description of the liver, thus providing more insight into drug distribution and underlying clearance processes. Likewise, in contrast to fractal models [11] translating the vascular branching to effective pharmacokinetics parameters, we consider the actual anatomical geometry of the organ and its vascular structures. A highly resolved representation is indispensable for models that can also describe individual, potentially heterogeneous, pathologies of the liver. One major drawback, however, of the spatially resolved model is the highly increased computational effort required to run the simulations, see Table 1 in Text S1.

To initially validate our spatially resolved model, we compared simulation results for spiramycin to experimental data obtained ex vivo with an isolated liver. The outflow concentrations simulated using the spatially resolved model and the experimental measurements in [32] are not in full agreement. Note, however, that the simulations of the isolated perfused liver are actually a prediction, since the original equations in the PBPK model were initially adjusted with respect to in vivo PK data [67]. In the light of this workflow it should be noted that the PBPK model represents only an intermediate step before the final spatially resolved model is ultimately established. It is only in this subsequent step that the liver model is integrated in the spatially resolved model, in this case to simulate ex vivo data from an isolated perfused liver [32]. Our approach hence extrapolates in vivo results obtained in a whole-body context to ex vivo data generated in an isolated liver as such supporting a structural transfer of knowledge. Hence, the setup of an isolated perfused liver is a suitable test case. The drawback of this prediction approach is the necessity of integrating experimental data coming from different sources which may partly explain the deviations in the stationary phase during the first 15 minutes during the onset of perfusion.

While deviations between experimental data and simulated concentrations can be attributed to large experimental standard deviations or limitations of in silico to ex vivo transferability, a general agreement between the spatially resolved model and experimental data can be observed (Figure 6). In particular, the clearance rate after the interruption of the spiramycin inflow is in good agreement with the experimental data. This illustrates how our spatially resolved model can be used to relate macroscopic observations in the plasma to distribution processes at the lobulus scale.

When applying the combined model to the case of steatosis it was found that already a spatially homogeneous change in the tissue composition leads to spatially heterogeneous differences in the distribution (Figure 8). The observed behavior showing the effect of an increased intracellular lipid content is actually a zonation effect on the length scale between terminal edges of the supplying and the draining vascular system. As discussed above, the qualitative result and the overall clearance are correct even though the flow distance between the two vascular trees is not the real hepatic lobule size. It was also found that the increased lipid content of the cells leads to longer intracellular retention times since the bound and thus immobile drug fraction increases. In turn, this leads to higher metabolization rates since lipid binding protects the compound from a fast wash-out due to increased retention times in steatotic livers.

Differences between spatially homogeneous and heterogeneous steatotic states were also analyzed (Figure 8). It was found that the difference in lipid accumulation between different lobes and within the lobes had an observable influence on the concentrations as retention times in the cellular subspace are longer in case of higher lipid accumulation. This heterogeneous effect is only visible in spatially resolved modeling (Figure 9). The model thus provides a mechanistic description of pathophysiological states of the liver and can moreover distinguish between different spatial patterns of the pathology.

Let us point out that both the temporal delay of the outflowing peak and differences between different steatotic states are inherent properties of the spatio-temporal model that the original compartmental PBPK model cannot describe. In contrast, the spatially resolved model can capture these effects in a qualitatively plausible way.

Our model predicts increased metabolization in steatotic livers, but decreased metabolization following 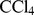-induced liver necrosis. The simulations hence provide testable predictions which can be compared to previously published experimental data [70], [71]. For steatotic livers, drug lipophilicity has been related to intrinsic elimination clearance in rats with nonalcoholic steatohepatitis (NASH) and control rats, respectively [70]. From this study, an increased clearance of approximately 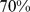 can be estimated for midazolam (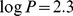) in steatotic animals. Even though this relationship has been established in rat livers perfused in situ and cannot be translated directly to our model, it nevertheless confirms qualitative validity of our simulations, since our model predicts an increased metabolization between 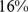 and 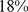. For more detailed comparisons, simulations and experimental measurements would need to be performed for the same experimental setup and in particular in the same species. However, the significantly increased clearance found experimentally in steatotic animals already points to the necessity of a more refined diseased model of steatosis since lipid accumulation alone is obviously not sufficient to explain the observed decrease in metabolic capacity. Possible model extension include, amongst others, previously discussed changes in microcirculation [72] and intracompartmental permeability [70]. For 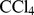-induced necrosis, our computational findings are also qualitatively validated by experimental observations, where a decreased metabolization of midazolam after 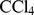 pretreatment has been found in rats [71]. Comparing the experimental findings with our current model structure indicates that decreased cytochrome levels in 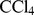-treated animals need to be considered as future model extensions. Here, our spatially resolved model could in particular be used to differentiate the contributions of enzymatic depletion and volumetric extension of necrosis on the decrease of metabolic capacity.

### Model Extensions

Despite the performance of the newly developed spatially resolved model, several limitations need to be addressed, which represent excellent opportunities for future model refinement. On the technical side, a more detailed geometric vascular model and flow simulation [73], not only using constant velocity in each cylinder could be considered. However, all this will drastically increase computational costs with little benefit as the intravascular flow patterns are largely irrelevant for what happens in the HHS. Deformations of the organ as in [24], [74] could also be taken into account. Likewise, changes of the effective blood viscosity [75] besides those due to the Fhrus-Lindqvist effect [44] could also be considered in the model.

The hepatic artery as the second supplying vascular system with other inflow concentration could become part of the model if its geometry and the local mixing of blood provided by portal vein and hepatic artery is known for the concrete situation considered [76]. This would also allow for more realistic flow velocities in the SVS. More generally, perfusion heterogeneity could also be considered as well as geometric scales of the perfusion [25]. A more detailed sensitivity analysis than merely one with respect to the vascular geometry (Figure 7 and Table 1 in Text S1) should be performed. For this purpose, known variations as well as measurement uncertainty of both PBPK model parameters and physiological/geometrical data need to be quantified, see e.g. [77]. For a physiologically relevant simulation output, such a sensitivity analysis will require substantial experimental and computational effort and should be part of a future study. Other implementations of the advection-PBPK simulation in the HHS should be investigated as well as the influence of computational resolution on the results. Comparing such fundamentally different implementations, however, is beyond the scope of this article.

When considering other metabolization processes, additional compounds, e.g. products formed by the metabolization or compounds only stored in the cellular HHS subspace can easily be included in the model. The exchange across membranes, 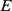 in Equation 7, can also be extended easily by active or other nonlinear processes.

As discussed above, comparing our computational simulations of pathophysiological states of the liver to experimental data [70], [71] suggests several model extensions. For steatosis, these include, but are not limited to, a significant increase in liver weight as observed in [59, Table 6] as well as changes and spatial variations in the effective permeability 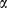 in Equation 4 and the volume fractions 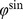, 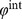, and 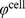, as a significant decrease of functional capillary density (sinusoidal length per area) was reported [59, Figure 17]. Sinusoidal flow velocities, however, were not observed to change significantly [59, Figure 16]. Other studies indicate that a change in the microcirculation should be taken into account in a more realistic model of steatosis, see [72]. Moreover, changes of the intracompartemental permeability [70, Figure 4A] as well as the activity of drug metabolizing enzymes due to steatosis as discussed in [78] may affect the cellular metabolization of compounds. For 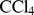-induced liver necrosis, changes in cytochrome levels [71] need to be considered in addition to necrotic changes in organ geometry. Here, our spatially-resolved model together with targeted liver histology could be used to differentiate between the different contributions to the decrease in metabolic capacity. Such integrative studies will allow further systematic analyses including iterative model testing and refinement in the future. More general pathological situations can be considered if one has solid knowledge of their spatial heterogeneity and their influence on the model parameters. In case of drugs being administered, also temporal changes of the parameters are possible and can be included in our model. A sensitivity analysis of the spatially resolved model with respect to such parameter perturbations could help to quantify their influence on the heterogeneity of drug distribution.

The model in general is not specific for mice, so it can be applied to other species provided the geometry information and PBPK parameters are available. Possibly other connectivity patterns between larger vascular structures and sinusoids depending on the species [79] (or, closely related in the simulation, diffusion of compounds through vascular walls) need to be taken into account. The vascular tree geometries used in the model are easily exchanged if more detailed experimentally [80] or algorithmically [42] determined data is available. Similarly, more detailed information about the geometric shape of lobuli (as in [81] for human livers) could be taken into account. In particular, in vivo imaging with a slightly higher level of detail than used here will allow running simulations for patient-specific vascular geometries, thus providing great promises for imaging and diagnostics in the future. Corrosion casts [82], or other types of ex vivo specimens, also scanned in micro-CT, provide higher resolution as time and high radiation doses are not an issue, but obviously do not permit in vivo imaging. Even higher resolution could be obtained by extracting vascular geometries from optical microscopy images of histological serial sections [80]. This, however, requires a tremendous experimental and image processing effort and again is not applicable in vivo.

### Outlook

As discussed above, possible zonation effects are qualitatively correctly observed at the length scale between the two incomplete vascular trees in our model rather than the actual length scale of hepatic lobuli. For correct observations in lobuli, our organ-scale simulations should be complemented by sinusoid-scale [83] or lobule-scale simulations in a multi-scale framework [21], [84].

Since the model can deal with pathological states of the liver and in particular spatially heterogeneous such states, their influence on the intrahepatic distribution of compounds could thereby be simulated pointing to future applications of spatio-temporal modeling in diagnostics. Here, comparison of our continuous simulations with new MRI or CT based image data could support the detection of pathological deviations. Predicting contrast agent distributions may help optimize time points for imaging after injection, benefiting from the much higher temporal and spatial resolution which our simulations can provide. The comparison of simulated and measured contrast agent distributions could therefore be used to identify changes in physiological parameters such that pathologies can be diagnosed.

The possibility to simulate heterogeneous distributions provides also important applications for the prediction of toxic side effects. The spatially resolved model allows a location-specific prediction of exposure profiles within the liver. PBPK models have been linked before to models at the cellular scale to predict toxicity responses within hepatic metabolism in response to paracetamol [21]. Together with the spatially resolved model, this can now be used to simultaneously simulate intralobular exposure profiles and the specific cellular response. This allows an in silico prediction of toxic side-effects following the drug administration during the first pass perfusion. Simulations of spatial heterogeneity can also be used to describe local zonation effects within an whole-organ context. PBPK models have been used before to describe genotype-specific differences in hepatic drug uptake [19] and intracellular metabolization [20]. Since the corresponding equations are also used in the spatially resolved model, it also becomes possible to describe first pass effects in a genotype-specific way.

Our spatially resolved model could be used for a wide range of technical and medical applications. It could for example be used for hypothermic machine perfusion [33] of livers to be transplanted for which mere static cold storage is ineffective. In this case, recirculation by a perfusion device needs to be considered, for which the influence on relevant compound concentrations can be described based on the existing PBPK models. Moreover, the model could be used to improve treatment planning for islet cell transplantations [34]. Here, mainly the perfusion simulation is needed to predict the distribution of a concentration of cells (not solutes) injected in the portal vein. Similarly, the model could help to improve intrahepatic injection of compounds, as discussed in the introduction. Another application could be optimization of targeted drug delivery [37] where drugs are injected in bound form and released at the desired location by mild hyperthermia induced by focused ultrasound. For this purpose, the model has to be combined with a heat transfer simulation [85].

For in vivo modeling within an organism context, more complicated full-body recirculation needs to be taken into account. This in turn requires our model to be integrated in whole-body simulations, regardless whether or not other organs are implemented at a comparable level of detail. Since our model has a spatially resolved internal state and (depending on the exchange and metabolization kinetics) may behave non-linearly, a transfer function approach [86] is not immediately applicable. Including recirculation in combination with our spatially resolved model will allow to mechanistically describe the distribution kinetics of fast acting drugs shortly after administration, similarly as it is has been done before with other circulatory models [11]. Extending such earlier approaches, our model will additionally use CT-based vascular trees within the liver.

While the spiramycin simulations above show general agreement with experimental results in [32], this is just a first step towards an exhaustive validation of our approach. Starting points for the important step of model validation in future studies could be comparing simulated and experimentally measured outflow concentrations similar to what was done in [32] or time-resolved imaging of the distribution of tracers (at least imaged on some slices; see e.g. [87]) for comparison to simulation results as in Figure 7. For the latter, also mean transit times [87] estimated from the results in Figure 6 or from Equation 6 in Text S1 could be used for comparison to experimental results. The setting of a compound not entering the cellular subspace, such as in [88] for MRI contrast agent in rats, could be a starting point with a simpler model. In both cases, the ex vivo setting potentially allows for artificially low and thus slow total perfusion, possibly enhancing CT or MR imaging at multiple time points. Much higher spatial resolution at a single time point could be obtained from histological whole-slide scans for which registration [89] and analysis [90] techniques are available. More generally, validation combined with a parameter sensitivity analysis could also help to narrow down parameter ranges where the model predicts physiologically realistic behavior. In this regard, our model could be used for experimental planning to estimate the required spatial and temporal resolution for imaging. Likewise, the number of animal sacrifices could be minimized by specific design of experiments. The model could furthermore be used to quantify the contribution of first pass effects to the overall bioavailability and the experimental variability.

As outlined above for steatosis and 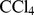-induced liver necrosis, our model can be used in combination with targeted experimental data to iteratively investigate pathological changes in liver physiology. Validation or falsification of computational predictions can thereby support mechanistic insights in underlying processes such that overall model structure can be adjusted accordingly. Due to the large level of detail included in our model, such modifications can be directly assigned to specific pathophysiological changes. It is thus possible to test hypotheses about the behavior of pathological livers or to analyze pharmacokinetic effects such as zonation [65]. To this end, PK data, which are ideally sampled densely in time both in the portal vein and in the hepatic vein need to be compared to specific simulation results. Experimentally, one could for example use isolated, pathological livers from genetically modified mice strains or use PBPK models to correlate plasma PK data in these animals with exposure profiles in the liver. Verifying or falsifying these in silico results can then, in turn, trigger further model refinement.

### Conclusion

We here present a novel method for spatially resolved simulations of first pass perfusion in the liver based on mass balance equations from physiologically based pharmacokinetic modeling as well as vascular geometries obtained by in vivo imaging. The spatio-temporal description of blood flow through the vascular systems in combination with distribution models used in pharmacokinetic modeling allows a mechanistic yet local description of compound perfusion within the tissue. Our combined model is capable of representing spatial parameter heterogeneity, so that the local impact of pathophysiological changes within the liver can be analyzed.

The model was used in the present study to investigate spatio-temporal effects of first pass perfusion for exemplary drugs. Two pathophysiological states, steatosis and 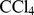-induced necrosis, were considered and were found to influence the distribution and metabolization of the compounds. Future applications of the model include optimized design of therapeutic treatments where spatially heterogeneous distributions or spatio-temporal perfusion effects are of relevance, e.g. targeted drug delivery, islet cell transplantations, or catheter placement for intrahepatic injections. We expect the spatially resolved model to be the foundation for further physiologically highly detailed modeling which will help to address specific spatial aspects of pharmacokinetics in the future.

## Supporting Information

Data S1 Datasets for the organ and vascular geometries used in the simulations including a basic 3D viewer using python and vtk.
(ZIP)

Text S1 Additional Figures; details on the discretization, implementation, and performance of the simulations.
(PDF)

Video S1 Video illustrating how the Constrained Constructive Optimization method generates vascular trees of desired level of detail, based on the experimentally obtained vascular trees.
(AVI)

Video S2 Video illustrating the vascular trees with 800 leaf nodes that was used for the simulations.
(AVI)

Video S3 Video showing (in real time) the distribution of the tracer CFDA SE during the first pass through our isolated mouse liver.
(AVI)
