# Supplementary material for: Spatio-Temporal Simulation of First Pass Drug Perfusion in the Liver
Source: PLoS Comput Biol. 2014 Mar 13;10(3):e1003499. doi: 10.1371/journal.pcbi.1003499 (PMC3952820; doi:10.1371/journal.pcbi.1003499)
Supplement: Text S1 — Additional Figures; details on the discretization, implementation, and performance of the simulations. (PDF) [file pcbi.1003499.s002.pdf]

Supporting Information: Text S1

Spatio-Temporal Simulation of First Pass Drug Perfusion in the Liver

Lars Ole Schwen   Markus Krauss   Christoph Niederalt   Felix Gremse
Fabian Kiessling   Andrea Schenk   Tobias Preusser   Lars Kuepfer

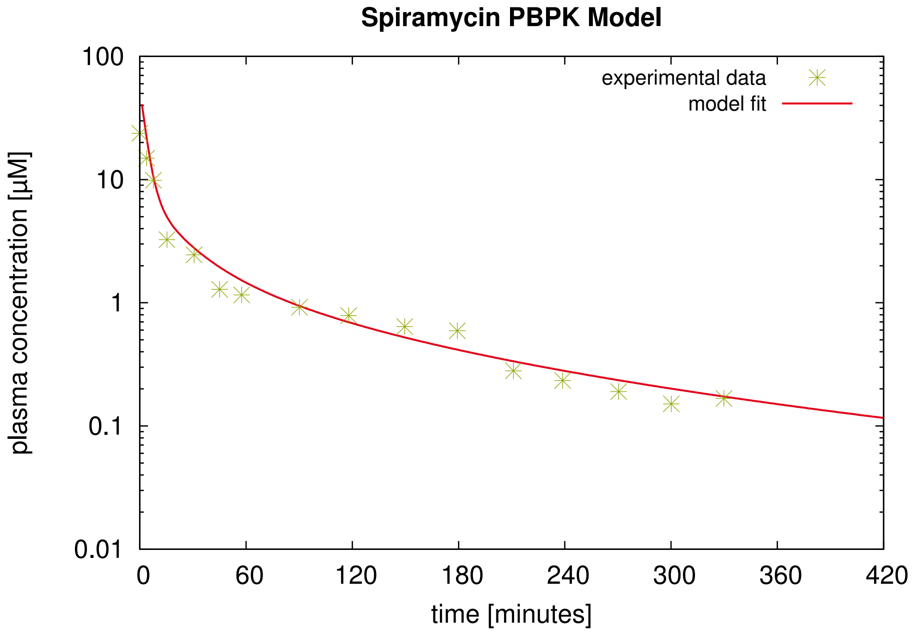

Figure 1: **Pharmacokinetics simulation for an intravenous administration of spiramycin.** Previously published PK data [1] for monkeys obtaining an intravenous dose of 50 mg spiramycin were used for basic model establishment. Intravenous PK data are necessary to identify systemic clearance capacity without overlaying processes in the gastro-intestinal tract during oral absorption. A linear hepatic metabolization term was considered in the model which was pre-parametrized using physicochemical compound parameters (MW, f_u, $\log P$).

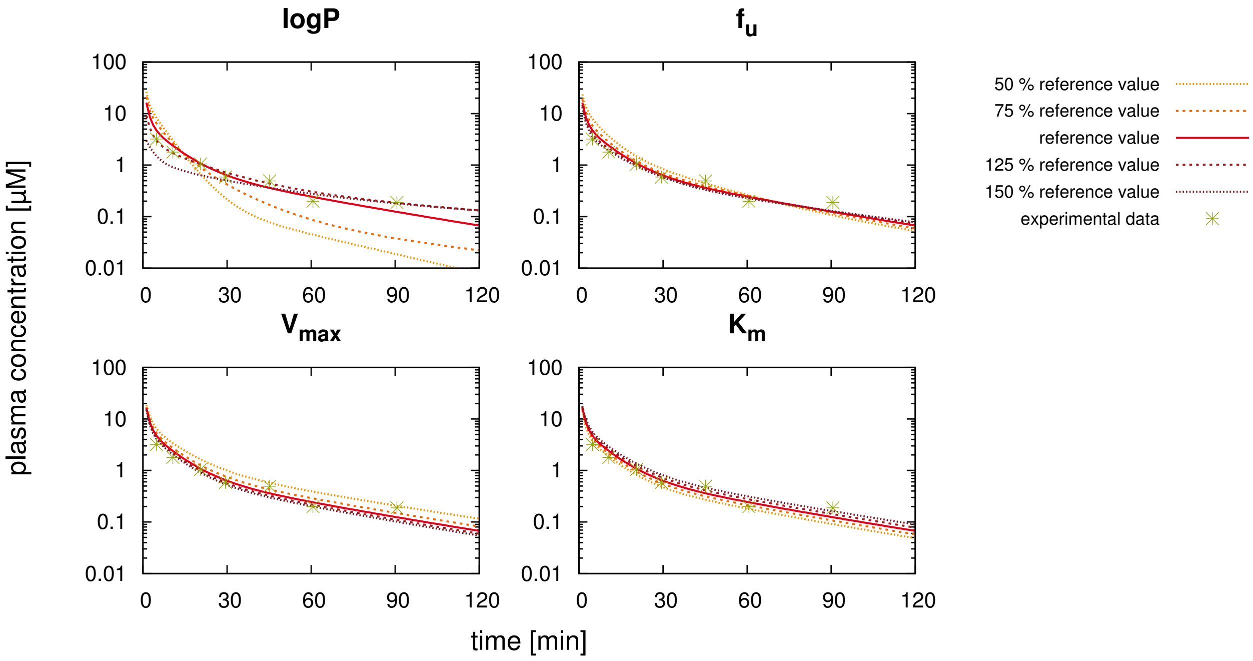

Figure 2: **Sensitivity analysis for the midazolam PBPK model.** The reference value of independent model parameters referring either to the physicochemistry of the drug ($\log P$, f_{u}) or the physiology of the organism (V_{max}, K_{m}) is perturbed to show its impact on the simulation behavior. Experimental PK data [2] are shown (stars) such that the simulation results (lines) can be compared to experimental observations.

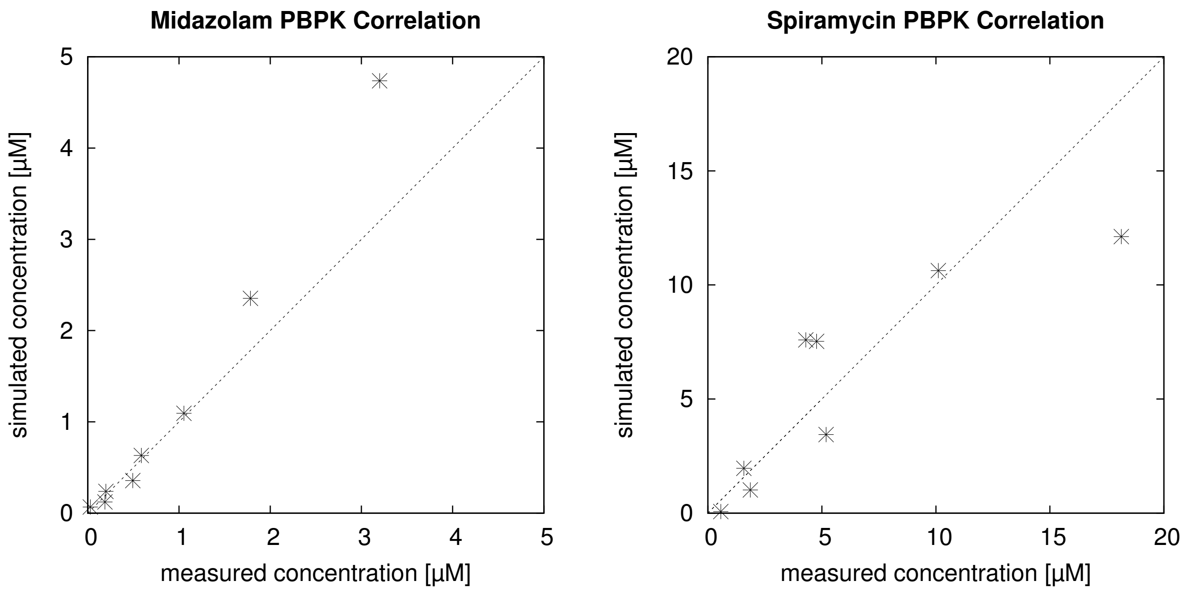

Figure 3: **Scatter plots illustrating the correlation between experimental and simulated concentration profiles.** The correlation for an intravenous dose of midazolam of 2.5 mg/kg (left, concordance correlation coefficient $\rho_c = 0.899$) and an oral dose of spiramycin of 400 mg/kg (right, concordance correlation coefficient $\rho_c = 0.845$) are shown [1,2].

1 Implementation of Advection in the Vascular Trees

For the discretization and implementation of the vascular advection model, we use a 1D ELLAM scheme going back to [3]. The PDE in Equation 2 in the article is written in weak form using special test functions ϑ chosen to be constant along the characteristic curves of Equation 2 in the article. An edge E discretized in space by equidistant grid points $x_j = x_0 + jh$ and in time by $t_k = t_0 + k\tau$ satisfying the condition

$$\frac{v\tau}{h} \leq 1 \tag{1}$$

i.e. the velocity may be at most the width of one grid cell per time step. Note that it is not necessary to satisfy a CFL condition [4] here, ELLAM can handle larger time steps, but it will be convenient to limit as we will see below. Basis functions for spatially discretizing the molar concentration profile are standard 1D piecewise affine-linear FE basis functions $\psi(x)$. The test functions ϑ are defined in terms of ψ as $\vartheta^k(x,t) = \psi(x - v(t - t_k))$, forming a partition of unity.

For the time interval $[t_k, t_{k+1}]$, the weak formulation also taking into account the cross-section area $A = A_\varnothing(E)$ can be stated as follows

$$\int_E A_\varnothing \int_{t_k}^{t_{k+1}} \big(- \partial_t c(x,t) - v\partial_x c(x,t)\big)\vartheta(x,t)\,\mathrm{d}t\,\mathrm{d}x = 0\,. \tag{2}$$

Substituting the spatial discretization $c(x,t_k) = \sum_j C_j^k \psi_j$ (same for $k+1$) and test functions $\vartheta_j^{k+1}(x,t) = \psi\left(x - v(t - t_{k+1})\right)$, we integrate by parts and observe that the space-time integral and other terms vanish due to the choice of ϑ being constant along characteristic curves. For the mathematical details omitted here, we refer to [3, Section 3] (with diffusivity $D = 0$). The system of equations for computing C_j^{k+1} in terms of C_j^k is hence

$$\begin{aligned} &C_{j-1}^{k+1} \int_{E^{k+1}} A_\varnothing \vartheta_j \psi_{j-1} + C_j^{k+1} \int_{E^{k+1}} A_\varnothing \vartheta_j \psi_j + C_{j+1}^{k+1} \int_{E^{k+1}} A_\varnothing \vartheta_j \psi_{j+1} \\ &= C_{j-2}^k \int_{E^k} A_\varnothing \vartheta_j \psi_{j-2} + C_{j-1}^k \int_{E^k} A_\varnothing \vartheta_j \psi_{j-1} + C_j^k \int_{E^k} A_\varnothing \vartheta_j \psi_j + C_{j+1}^k \int_{E^k} A_\varnothing \vartheta_j \psi_{j+1} \end{aligned} \tag{3}$$

$E^m = E \times \{t_m\}$ and terms for $j-2$ and $j \pm 1$ only present if the indices are in the respective range. Inflow and outflow at the spatial boundary will be discussed below. This results in a system of equations of the form

$$MC^{k+1} = TC^k \tag{4}$$

with M being a finite element mass matrix the cross-section area $A_\varnothing$) and a quadridiagonal matrix T with one upper and two lower diagonals. In fact, we use a lumped mass matrix M for reasons discussed at the end of this section.

Note that the factor $A_\varnothing$ in Equation 2 does not make a difference when only considering a single edge as it appears on both sides of Equation 4, but will come in handy when we now consider branchings because it automatically takes care of mass conservation of the compounds considered. Moreover, let us point out that we always index grid points such that indices increase along the direction of flow.

Inflow Boundary Conditions. Inflow boundary conditions (depending on time) are mapped to spatial data on a virtual extension of the corresponding edge by one grid cell in upflow direction, essentially resulting in an additional term on the right hand side of Equation 4.

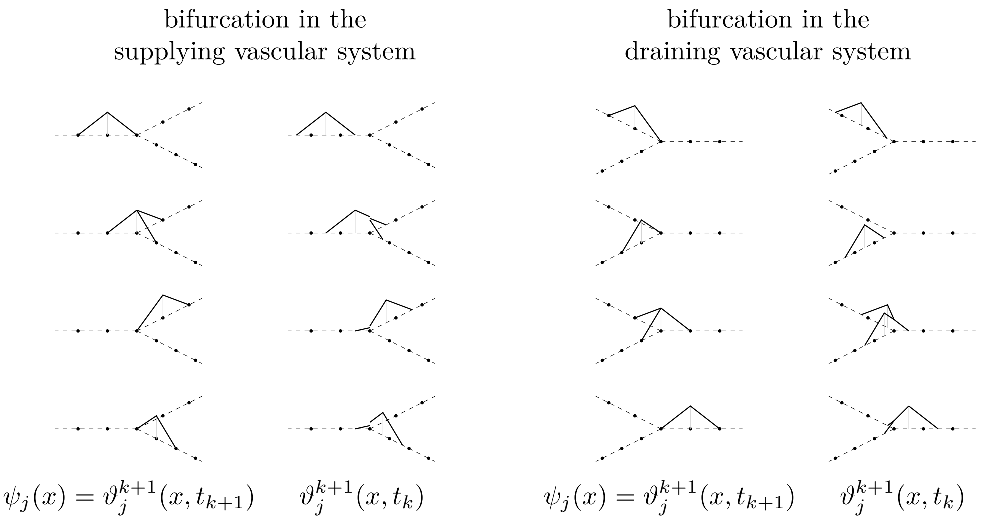

Figure 4: **Visualization of ELLAM basis functions ψ_j and test functions ϑ_j near bifurcations of the supplying and draining vascular systems.** Notice the duality between continuous splitting of molar concentrations in the supplying vascular system and discontinuous test function $\vartheta_j^{k+1}(\cdot, t_k)$ after tracing back, vice versa for the draining vascular system. (Figure adapted from [5, Figures 4.7, 4.8, 4.10, and 4.11].)

Branching Points. As for branching points, let us consider the case of the supplying vascular system first. In this case, the end point of parent edge coincides with the start points of the daughter edges, only one degree of freedom (DOF) as part of the parent edge is assigned to that point. Basis functions ψ_b corresponding to such DOF are supported in multiple edges. Test functions $\vartheta_\mathrm{b}^{k+1}(x, t_{k+1})$ at the new time step still coincide with the corresponding ψ_b, tracing them back along characteristics results in discontinuous $\vartheta_\mathrm{b}^{k+1}(x, t_k)$ with modified slope on the upflow (i.e. parent) edge due to the discontinuous velocity, see Figure 4. Let us point out that, despite the sketch showing only a bifurcation, this scheme is easily adapted to multifurcations.

In case of the draining vascular system, the end points of daughter edges coincide with the start points of the parent edge. Basis functions ψ_b are the same as before, test functions at the old time step coincide with the corresponding ψ_b. However, tracing them back in upflow direction now results in continuous test function $\vartheta_\mathrm{b}^{k+1}(x, t_k)$ at the old time step, again with different slope on the upflow (this time daughter) edges, see Figure 4.

Grid spacings h for the different edges need not be the same (which is not even possible because edges have arbitrary length and are to be discretized with an integer number of grid points), but the time step is a common one. If we require at least four grid points per edge and Equation 1 for each edge, at most one bifurcation needs to be considered for adapting the ψ and ϑ. This is one reason for the restriction in Equation 1.

In the analog of Equation 2, we now need to integrate over multiple edges E and take into account the respective cross-section area $A_\varnothing = A_\varnothing(E)$. When considering a whole vascular tree, the value vectors C^k in Equation 4 then have block structure with one block per vascular edge. The corresponding analogs of matrices M and T in Equation 4 also have a block structure, mainly consisting of individual such M and T on the diagonal blocks with individual additional contributions from ψ and ϑ supported on two adjacent edges. For a more detailed discussion of these couplings, we refer to [5, Section 4.3].

Another observation in [5, Section 4.3] is that using Equation 4 may lead to artificial oscillations at draining bifurcations introducing over- and undershoots, i.e. not preserving the ranges of the molar concentrations. This well-known effect both ELLAM simulations can be remedied [6] by

lumping M with the additional benefit of only needing to invert a diagonal matrix but the drawback of introducing artificial diffusion due to the numerical scheme.

2 Implementation of Advection in the Homogenized Hepatic Space

Discretization of Equation 5 in the article leads to an analog of Equation 4 with a 3D finite element mass matrix M and a matrix T on the right hand side with a more general sparsity structure. Again and for the same reasons as in the 1D case, lumped M was used.

Test functions ϑ for the ELLAM formulation are obtained from standard multilinear FE tent functions ψ as before by tracing back along the characteristics. This is more technical than in the 1D case because any upflow direction is possible now and quadrature in 3D needs to distinguish more possible cases of overlap of supports of ϑ and ψ, also resulting in a more complicated sparsity structure of the resulting matrix T. Similar to Equation 1 in the 1D case, we again restrict the maximal time step so that the maximal velocity is smaller than one grid cell per time step.

Given the discretization of the vascular trees using 1D piecewise linear basis functions and the HHS using piecewise trilinear basis functions, the source terms can be treated by matrix–vector multiplication. For this purpose, the line integrals over terminal edges of products of the two types of basis functions need to be evaluated. As the product is a fourth-order polynomial, we use Gauss quadrature with 3 points [7] providing fifth-order accuracy. Next to bifurcations where the value is stored for the parent edge, this would require interpolation between values stored for different edges. As a slight simplification and reduction of technicalities, we consider piecewise constant basis functions along the edges and assemble the matrix accordingly.

Discrete velocity profiles as the one computed here are not exactly conservative, i.e. have vanishing divergence away from sources and sinks, but this effect can be compensated for [6, Section 5]. Additionally, we are dealing with lower-dimensional sources and sinks, making it necessary to compensate for discretization artifacts.

0. As a preprocessing step, we start with an initial concentration of constant 1 in the HHS and the vascular systems with the same value 1 for the inflow to the supplying vascular system. Computing one coupled time step for the vascular system and HHS advection with appropriate source and sink terms followed by Gaussian diffusion by $1/\sqrt{2}$ voxels [8], we determine an additive correction for the HHS advection step.

1. In each simulation time step, we first perform the advection time step in the vascular systems and compute the amount supplied to and drained from the HHS.

2. Next we compute sources (according to the values in the supplying vascular system) and sinks (according to values in the HHS near the terminal edges of the draining vascular system) and perform the HHS advection time step, followed by Gaussian diffusion by $1/\sqrt{2}$ voxels. We then apply the correction term from step 0, locally scaled to fit the current molar concentration values. After a second diffusion step by the same amount as before, we compare the update of the contained amount in the HHS to the amount balance of step 1 and rescale accordingly to satisfy conservation of molar amounts.

3. Finally, we compute the PBPK time step in the HHS which may change molar amounts by design as it represents not only exchange but also metabolization. Here, we merely impose a lower limit of 0 for the molar concentrations in case the numerical scheme has computed slightly negative values.

In the literature, more advanced ELLAM schemes have been presented. Instead of separating advection and PBPK time steps, the PBPK could be linearized as in [9] or Runge–Kutta-based

higher order time stepping can be used [10]. However, for more involved PBPK terms, it is not straight-forward to estimate the influence of linearization and a decoupling of the time stepping for the two processes permits a more efficient implementation. For a more detailed discussion of spatially varying velocities, we refer to [11], the latter also presenting a multiscale ELLAM approach, and [12] for a numerical upscaling procedure.

Unstructured meshes have also been used in ELLAM simulations [13]. This approach could help resolve the velocity variations in the HHS near the exchange with vascular edges, but at the well-known cost of a less efficient implementation than the structured hexahedral meshes we use. This trade-off could be investigated in a further study.

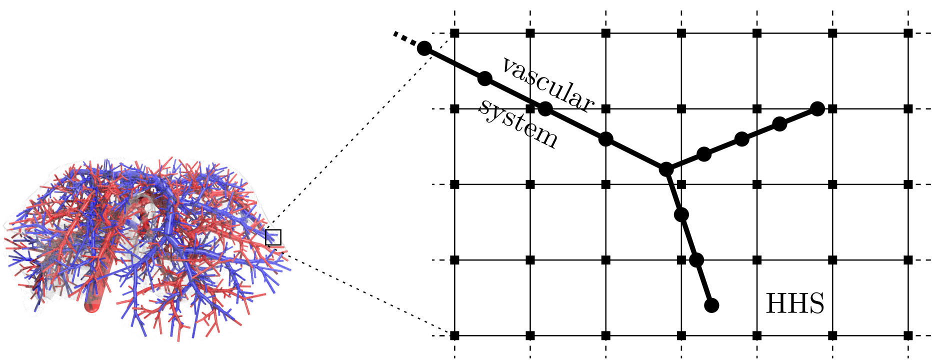

Figure 5: **Sketch of grids for spatial discretization.** The HHS is spatially discretized by a regular hexahedral computational grid (shown in 2D). The edge-wise equidistant 1D discretization of the vascular systems is chosen independently.

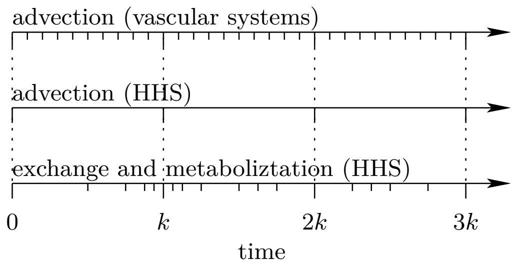

Figure 6: **Sketch of the time steps used for temporal discretization.** For the temporal discretization, independent time stepping schemes for three processes are used alternatingly with synchronization after a fixed time step k.

3 Performance of the Perfusion-Storage Model

Simulations using the CFDA SE parameters were also used to (a) investigate how the level of detail in the vascular structures influence the resulting outflow concentrations and (b) to assess the computational performance. The datasets used for this comparison are available in Data S1.

Figure 7 shows that about 800 leaf nodes in the trees are sufficient to avoid artifacts due to insufficient resolution such as flow heterogeneities. Higher numbers of leaf nodes only change the outflow concentration profile by a minor additional delay, but at the cost of increasing computational effort, see Table 1.

Computational Performance. The simulations can be run on a standard desktop PC, CPU times for the CFDA SE simulations as described above are reported in Table 1 for a Intel Xeon X7550 2.0 GHz CPU. For 800 leaf nodes, the spatially resolved simulation involves the two coupled advection simulations (actually being the largest portion of the workload) in addition to 49 114 instances of the PBPK ordinary differential equation in the HHS. Compared to a four-subcompartment simulation without spatial resolution, the computation time increases only by a factor of 160 241. Simulations were only run single-threaded, first test show a moderate gain in performance by parallelizing the code using OpenMP. However, for the resolution described above, we are far from real-time simulations.

Typical Transit Times. As we are not using the full vascular trees, we need to discuss the influence of incompletely resolved vascular trees on the velocity profile and the time a given metabolite takes to pass through the HHS. For assessing this, let

$$d_{\text{typ}} := \underset{e_s \text{ terminal edge of SVS}}{\text{mean}} \; \underset{e_d \text{ terminal edge of DVS}}{\min} \text{dist}\left(\text{midpoint}(e_s), \text{midpoint}(e_d)\right) \tag{5}$$

be the average distance between midpoints of terminal edges of the supplying vascular system to the closest midpoint of edges of the draining vascular system, a typical distance the blood needs to pass through the HHS. Moreover, let $v_{\text{avg}} := \frac{1}{\text{Volume(HHS)}} \int_{\text{HHS}} \|v\|$ be the average Euclidean norm of the velocity, so that we can define a *typical transit time*

$$t_{\text{typ}} := \frac{d_{\text{typ}}}{v_{\text{avg}}} \tag{6}$$

for the blood through the HHS. We refer to [14] for an overview of other notions of transit times for metabolites in the liver.

The amount of blood flowing between the vascular structures per unit time is independent of the level of detail in the vascular structures, merely the flow through each terminal edge changes. If the perfusion of the liver volume is essentially homogeneous, we obtain

$$t_{\text{typ}} \approx \frac{\text{liver volume} \cdot \text{porosity}}{\text{total perfusion}} = \frac{\text{Volume(HHS)} \cdot \varphi^{\text{sin}}}{Q_{\text{tot}}} := t_{\text{perf}} \tag{7}$$

independent of the concrete vascular geometries. In particular, it is independent of the level of detail in the vascular structures, provided the perfusion is sufficiently homogeneous. With Volume(HHS) $= 1.077\,\text{ml}$, $\varphi^{\text{sin}} = 0.104$, and $Q_{\text{tot}} = 2.1\,\text{ml/min}$ as above, we obtain $t_{\text{perf}} = 3.200\,\text{s}$. This estimate indeed matches the values t_{typ} obtained for different vascular resolutions in Table 1.

Let us point out that the time for passing through the vascular structures of course depends on how much detail is contained in them. Thus the total transit time of a concentration depends on the vascular resolution. However, no metabolization takes place inside the vascular structures in our model, so only t_{typ} or t_{perf} matter for the total metabolization.

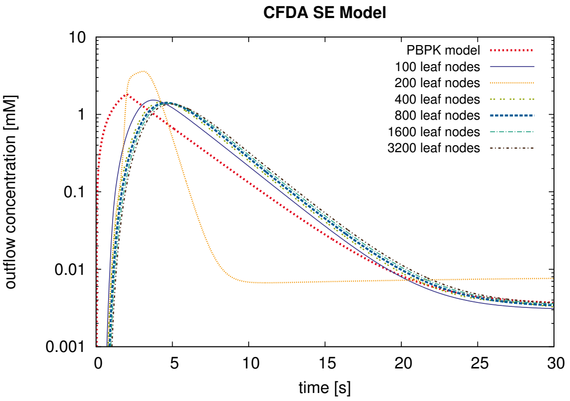

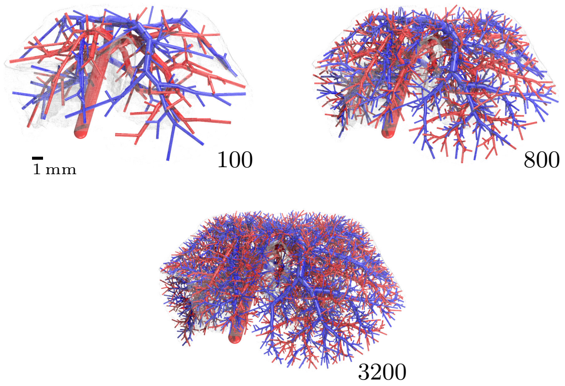

Figure 7: **Influence of vascular level on detail on the simulation results.** The plot *(left)* shows outflow concentrations obtained by our CFDA SE simulations in the healthy state for vascular trees in the isolated mouse liver with different level of detail (number of leaf nodes for the supplying and draining vascular systems each). For less than 800 leaf nodes, results differ notably while more than 800 leaf nodes do not lead to big changes. Four of the vascular structures with different number of leaf nodes per tree are visualized *(right)*.

number of leaf nodes	100	200	400	800	1600	3200
# leaf nodes used in SVS	100	200	400	799	1595	3182
# leaf nodes used in DVS	100	200	400	800	1599	3184
typical transit time [s]	3.061	2.991	3.052	3.050	3.048	3.074
relative mass lost [10^{-9}]	6.114	11.30	6.662	7.101	7.323	6.830
CPU time factor [1]	181.22	224.79	202.77	269.68	450.22	1181.86
memory consumption [MiB]	402	409	427	493	728	1501

Table 1: **Influence of vascular level of detail on transit times, mass conservation and computational performance.** For a computational resolution of 280 µm, the table shows the influence of the vascular resolution (number of leaf nodes) in the isolated mouse liver model on computational parameters for the CFDA SE simulation. Due to a minimum-length requirement for considering terminal edges in the simulations, some terminal edges remain unused for the finer trees. The estimated typical transit time is essentially independent of vascular resolution. Mass conservation after 30 s is also satisfied independent of the vascular resolution up to a factor of about 10^{-8}.

4 Software Architecture

In this Section, we give an overview of our implementation of the simulations. Using C++ notation, we present the main structure of the class structure, omitting most of the technical details. In particular, all the technicalities implementing ELLAM for the 1D and 3D advection are left out. The 1D case is described in [5, Section 4], our implementation here is based on the corresponding code available as part of [15].

4.1 Main Program

```
int main ( const int, const char** const argv ) {
  Parameters params;
  loadParameters ( argv[1], params );
  OrganVascularGeometry ovg;
  loadOrganAndVascularTrees ( params, ovg );
  DrugMetabolizationReactionBase *pReac = NULL;
  switch ( params.drug ) {
  case CFDA_SE:
    pReac = new MetabolizationReactionCFDASE ( /* ... */ );
    /* ... */
  }
  InflowTimeCurve inflowTimeCurve;
  // set up bolus profile for inflowTimeCurve

  HHSFlowSimulator hhsFlowSimulator ( params, ovg, inflowTimeCurve, *pReac );

  VascularConcVector
    vascConcVecSVS ( ovg._supplyingVS, params.numSubstVasc ),
    vascConcVecDVS ( ovg._drainingVS, params.numSubstVasc );
  HHSConcVector hhsConcVec ( ovg._organMask, params.numSubstHHS );

  for ( unsigned int ts = 0;
        ( ts * params.timeStepSynchro ) < params.timeToSimulate;
        ++ts ) {
    hhsFlowSimulator.doTimeStep ( ts * params.timeStepSynchro,
                                  vascConcVecSVS,
                                  vascConcVecDVS,
                                  hhsConcVec );
    hhsFlowSimulator.outputForPlotting ( ts * params.timeStepSynchro,
                                         vascConcVecSVS,
                                         vascConcVecDVS,
                                         hhsConcVec );
  }
  delete ( pReac );
  return ( EXIT_SUCCESS );
}
```

4.2 Simulation Classes

```
class HHSFlowSimulator {
public:
  HHSFlowSimulator ( const Parameters &Params,
                     const OrganVascularGeometry &Ovg,
                     const InflowTimeCurve &InflowTimeCurve,
                     const DrugMetabolizationReactionBase &MetabReac )
  : _params ( Params ), _ovg ( Ovg ), _inflowTimeCurve ( InflowTimeCurve ),
    _exchangeMetabolizationTimeStepper ( MetabReac, Params.timeStepSynchro ) {
    // determine _hhsFlowVelocities
  }

  void doTimeStep ( const double Time,
                    VascularConcVector &VascConcVecSVS,
                    VascularConcVector &VascConcVecDVS,
                    HHSConcVector &HHSConcVec ) {
    std::vector< HHSSourceSink > transferredMass ( _params.numSubstVasc );
    // easily parallelized using OpenMP
    for ( unsigned short s = 0; s < _params.numSubstVasc; ++s ) {
      advectionTimeStepVascSys ( VascConcVecSVS[s], VascConcVecDVS[s],
                                 HHSConcVec[s], transferredMass[s] );
    }
    // easily parallelized using OpenMP
    for ( unsigned short s = 0; s < _params.numSubstHHS; ++s ) {
      advectionTimeStepHHS ( transferredMass[s], HHSConcVec[s] );
    }
    exchangeAndMetabolizationTimeStep ( HHSConcVec );
  }

  void outputForPlotting ( const double Time,
                           const VascularConcVector &VascConcVecSVS,
                           const VascularConcVector &VascConcVecDVS,
                           const HHSConcVector &HHSConcVec ) const {
    /* ... */
  }

protected:
  void advectionTimeStepVascSys ( VascularConc &VascConcSVS,
                                  VascularConc &VascConcDVS,
                                  const HHSConc &HHSConc,
                                  HHSSourceSink &TransferredMass ) {
    // do advection sub time steps for SVS (using _inflowTimeCurve),
    // computing source part in TransferredMass
    //
    // do advection sub time steps for DVS (accumulating _outflowTimeCurve),
    // computing sink part in TransferredMass
  }

  void advectionTimeStepHHS ( const HHSSourceSink &TransferredMass,
                              HHSConc &HHSConc ) const {
    // do advection sub time steps for HHS
    // compensate for non-vanishing divergence of
    // velocity vector field to ensure conservation of molar amounts
  }

  void exchangeAndMetabolizationTimeStep ( HHSConcVector &HHSConcVec ) const {
    _exchangeMetabolizationTimeStepper.doTimeStep ( HHSConcVec );
  }

private:
  const Parameters            &_params;
  const OrganVascularGeometry &_ovg;
  const InflowTimeCurve       &_inflowTimeCurve;  // total model inflow (given)
  OutflowTimeCurve             _outflowTimeCurve;
                              // total model outflow (computed during time steps)
  HHSVelocityVectorField       _hhsFlowVelocities;
  RKF45TimeStepping           _exchangeMetabolizationTimeStepper;
  /* ... */
};
```

```
class DrugMetabolizationReactionBase {
  virtual void evaluateDerivative ( /* ... */ ) const = 0;
};

class MetabolizationReactionCFDASE : public DrugMetabolizationReactionBase {
public:
  /* ... */
  void evaluateDerivative ( /* ... */ ) const { /* ... */ }
};

class MetabolizationReactionMidazolam : public DrugMetabolizationReactionBase {
public:
  /* ... */
  void evaluateDerivative ( /* ... */ ) const { /* ... */ }
};

class RKF45TimeStepping {
public:
  RKF45TimeStepping ( const DrugMetabolizationReactionBase &MetabReac,
                      double TimeStep )
  : _metabReac ( MetabReac ), _timeStep ( TimeStep ) {
    /* ... */
  }
  void doTimeStep ( HHSConcVector& hhsConcVec ) const {
    // for each grid point in the HHS
    //   do RKF45 sub time steps up to _timeStep
  }

private:
  const DrugMetabolizationReactionBase &_metabReac;
  const double _timeStep;
  /* ... */
};
```

4.3 Data Structures and Utilities

```
struct OrganVascularGeometry {
  OrganMask _organMask;
  VascularSystem _supplyingVS, _drainingVS;
};

void loadParameters ( const char* const ParameterFileName, Parameters &Params ) {
  // load Params from ParameterFileName
}

void loadOrganAndVascularTrees ( Parameters &Params, OrganVascularGeometry &Ovg ) {
  // load organ mask and vascular geometry from files
}
```

References

[1] Chew WK, Segarra I, Ambu S, Mak JW (2012) Significant reduction of brain cysts caused by toxoplasma gondii after treatment with spiramycin coadministered with metronidazole in a mouse model of chronic toxoplasmosis. Antimicrobial agents and chemotherapy 56: 1762–1768. doi:10.1128/AAC.05183-11.

[2] Kuze J, Mutoh T, Takenaka T, Morisaki K, Nakura H, et al. (2009) Separate evaluation of intestinal and hepatic metabolism of three benzodiazepines in rats with cannulated portal and jugular veins: comparison with the profile in non-cannulated mice. Xenobiotica 39: 871–880. doi:10.3109/00498250903215382.

[3] Celia MA, Russell TF, Herrera I, Ewing RE (1990) An Eulerian-Lagrangian localized adjoint method for the advection-diffusion equation. Advances in Water Resources 13: 187–206. doi:10.1016/0309-1708(90)90041-2.

[4] Courant R, Friedrichs K, Levy H (1928) Über die partiellen Differenzengleichungen der Mathematischen Physik. Mathematische Annalen 100: 32–74. doi:10.1007/BF01448839.

[5] Schwen LO (2005) Numerical Simulation of Transport and Diffusion in Drainage Media. Diplom thesis, University of Duisburg-Essen.

[6] Neubauer T, Bastian P (2005) On a monotonicity preserving Eulerian–Lagrangian localized adjoint method for advection–diffusion equations. Advances in Water Resources 28: 1292–1309. doi:10.1016/j.advwatres.2005.04.010.

[7] Abramowitz M, Stegun IA, editors (1972) Handbook of Mathematical Functions With Formulas, Graphs, and Mathematical Tables. National Bureau of Standards, 10th edition.

[8] Aubert G, Kornprobst P (2006) Mathematical Problems in Image Processing, Springer, volume 147 of *Applied Mathematical Sciences*, chapter Image Restoration. pp. 65–147. doi:10.1007/978-0-387-44588-5.

[9] Våg JE, Wang H, Dahle HK (1996) Eulerian–Lagrangian localized adjoint methods for systems of nonlinear advective–diffusive–reactive transport equations. Advances in Water Resources 19: 297–315. doi:10.1016/0309-1708(96)00006-1.

[10] Wang H, Al-Lawatia M, Telyakovskiy AS (1997) Runge–Kutta characteristic methods for first-order linear hyperbolic equations. Numerical Methods for Partial Differential Equations 13: 617–661. doi:10.1002/(SICI)1098-2426(199711)13:6<617::AID-NUM3>3.0.CO;2-U.

[11] Cheng A, Wang K, Wang H (2010) A preliminary study on multiscale ELLAM schemes for transient advection-diffusion equations. Numerical Methods for Partial Differential Equations 26: 1405–1419. doi:10.1002/num.20496.

[12] Arbogast T (2000) Numerical Treatment of Multiphase Flows in Porous Media, Springer, volume 552 of *Lecture Notes in Physics*, chapter Numerical Subgrid Upscaling of Two-Phase Flow in Porous Media. pp. 35–49. doi:10.1007/3-540-45467-5_3.

[13] Younes A, Ackerer P (2005) Solving the advection-diffusion equation with the Eulerian–Lagrangian localized adjoint method on unstructured meshes and non uniform time stepping. Journal of Computational Physics 208: 384–402. doi:10.1016/j.jcp.2005.02.019.

[14] Mellick GD, Anissimov YG, Bracken AJ, Roberts MS (1997) Metabolite mean transit times in the liver as predicted by various models of hepatic elimination. Journal of Pharmacokinetics and Biopharmaceutics 25: 477–505. doi:10.1023/A:1025797126763.

[15] AG Rumpf, Institute for Numerical Simulation, University of Bonn (2011). Quocmesh software library, version 1.3. URL http://numod.ins.uni-bonn.de/software/quocmesh/index.html.
